# Machine learning prediction of motor function in chronic stroke patients: a systematic review and meta-analysis

**DOI:** 10.3389/fneur.2023.1039794

**Published:** 2023-06-13

**Authors:** Qinglin Li, Lei Chi, Weiying Zhao, Lei Wu, Chuanxu Jiao, Xue Zheng, Kaiyue Zhang, Xiaoning Li

**Affiliations:** ^1^Second Clinical Medical School, Heilongjiang University of Chinese Medicine, Harbin, Heilongjiang, China; ^2^Department of Acupuncture, The Second Affiliated Hospital of Heilongjiang University of Chinese Medicine, Harbin, Heilongjiang, China; ^3^Department of Acupuncture, The Third Affiliated Hospital of Zhejiang Chinese Medical University, Hangzhou, Zhejiang, China; ^4^Department of Neurorehabilitation, Taizhou Enze Medical Center Luqiao Hospital, Taizhou, Zhejiang, China

**Keywords:** machine learning, model prediction, stroke, motor function, systematic review

## Abstract

**Background:**

Recent studies have reported that machine learning (ML), with a relatively strong capacity for processing non-linear data and adaptive ability, could improve the accuracy and efficiency of prediction. The article summarizes the published studies on ML models that predict motor function 3–6 months post-stroke.

**Methods:**

A systematic literature search was conducted in PubMed, Embase, Cochorane and Web of Science as of April 3, 2023 for studies on ML prediction of motor function in stroke patients. The quality of the literature was assessed using the Prediction model Risk Of Bias Assessment Tool (PROBAST). A random-effects model was preferred for meta-analysis using R4.2.0 because of the different variables and parameters.

**Results:**

A total of 44 studies were included in this meta-analysis, involving 72,368 patients and 136 models. Models were categorized into subgroups according to the predicted outcome Modified Rankin Scale cut-off value and whether they were constructed based on radiomics. C-statistics, sensitivity, and specificity were calculated. The random-effects model showed that the C-statistics of all models were 0.81 (95% CI: 0.79; 0.83) in the training set and 0.82 (95% CI: 0.80; 0.85) in the validation set. According to different Modified Rankin Scale cut-off values, C-statistics of ML models predicting Modified Rankin Scale>2(used most widely) in stroke patients were 0.81 (95% CI: 0.78; 0.84) in the training set, and 0.84 (95% CI: 0.81; 0.87) in the validation set. C-statistics of radiomics-based ML models in the training set and validation set were 0.81 (95% CI: 0.78; 0.84) and 0.87 (95% CI: 0.83; 0.90), respectively.

**Conclusion:**

ML can be used as an assessment tool for predicting the motor function in patients with 3–6 months of post-stroke. Additionally, the study found that ML models with radiomics as a predictive variable were also demonstrated to have good predictive capabilities. This systematic review provides valuable guidance for the future optimization of ML prediction systems that predict poor motor outcomes in stroke patients.

**Systematic review registration:**

https://www.crd.york.ac.uk/prospero/display_record.php?ID=CRD42022335260, identifier: CRD42022335260.

## 1. Introduction

Stroke is an acute cerebrovascular disease caused by sudden rupture of intracranial vessels or vascular obstruction preventing blood from flowing into the brain and thereby leading to brain tissue damage. Based on its pathological pattern, stroke can be classified into ischemic stroke (IS) and hemorrhagic stroke (HS). The Global Burden of Diseases, Injuries, and Risk Factors Study 2017 (GBD 2017) reported that stroke resulted in 6.17 million deaths and is the second leading cause of death and disability worldwide (1). According to the 2021 Guideline for the Prevention of Stroke in Patients With Stroke and Transient Ischemic Attack From the American Stroke Association (ASA), high blood pressure, diet, abdominal obesity, physical inactivity and smoking represent 82% (2) of the population-attributable risk (PAR) in patients with IS and HS. Although most IS patients have received effective treatments, many of them still suffer certain functional impairment after treatment. Motor function outcome in stroke survivors, as a primary determinant of the burden of stroke, directly determines their quality of life. Furthermore, physical disability is a key factor for the occurrence of mental disorders, such as depression, which occurs in 33% of stroke survivors (3). Therefore, the motor function-related outcome is one of the greatest concerns for stroke patients and their families. Clinically, it is extremely significant for clinicians to judge the prognosis of stroke patients and make a long-term treatment plan for those with a poor motor function outcome (4).

With rapid advances in medical and health informatization, medical data in a larger scale can be divided into more types, and the health care field has also entered a new era of big data. Due to the large scale, diversified types and high hidden value of medical data, ML algorithms have been widely used in the medical field (5–8). ML can be defined as a subfield of artificial intelligence (AI) that uses computerized algorithms to automatically improve performance through an iterative learning process or experience (i.e., data collection) (9). Different from traditional prediction models that use selected variables for calculation, ML techniques can easily incorporate a large number of variables to describe the complex and unpredictable nature of human physiology in a clearer way. Therefore, ML may be helpful for clinical prediction and identification of new prognostic markers (10). In recent years, many ML methods have been applied to the diagnosis and assessment of stroke (11, 12), including the evaluation of stroke severity (13), analysis of cerebral edema (14), prediction of hematoma expansion (15), and incidence prediction (16). Therefore, ML model predictions not only aid in disease analysis, prevention, diagnosis, and patient monitoring, but also help clinicians handle massive amounts of data in a more accurate and efficient manner (17).

In the literature, the published systematic reviews lack ML model prediction analysis of motor function 3–6 months post-stroke, especially regarding model predictive capabilities in different outcome cut-off values to accurately determine efficacy of ML predictions. Additionally, individual original studies may not be able to statistically assess the robustness of prediction results. Therefore, this systematic review and meta-analysis was conducted to assess the performance of current ML models as clinical tools for predicting medium- and long-term recovery of motor function in stroke patients.

## 2. Methods

This systematic review and meta-analysis was performed in accordance with the Preferred Reporting Items for Systematic Reviews and Meta-Analyses 2020 (PRISMA 2020), and prospectively registered in PROSPERO (CRD42022335260).

### 2.1. Search strategy

A comprehensive and systematic search was conducted in PubMed, Embase, Cochorane, and Web of Science databases. The retrieval was as of April 3, 2023. A researcher (Xiaoning Li) designed the keywords and search strategy of this systematic review, and both subject headings and free words were searched. The complete search strategy can be found in the Supplementary Table S1.

### 2.2. Inclusion and exclusion criteria

#### 2.2.1. Inclusion criteria

(1) Patients were diagnosed with IS or HS on CT or MRI. IS included large vessel occlusion, anterior circulation infarction and posterior circulation infarction. (2) ML was used to predict motor function in patients 3–6 months post-stroke.

(3) The Modified Rankin Scale was used as the outcome measure. (4) Aged 18 and older. (5) Articles written in English or translated into English. (6) Randomized controlled trials, cohort studies, case-control studies.

#### 2.2.2. Exclusion criteria

(1) Patients were clumsy in physical activities or unable to function independently before stroke (Premorbid Modified Rankin Scale ≥ 2). (2) Cerebral hemorrhage resulted from secondary causes, such as cerebral trauma and subarachnoid hemorrhage. (3) Prediction models applied clinical scoring rather than ML. (4) Case reports, protocols, editorials, and perspectives that have no original data.

### 2.3. Literature screening and data extraction

All of the retrieved studies were imported into Endnote for management. After automatic and manual removal of duplicates, two researchers (Weiying Zhao and Xue Zheng) independently assessed remaining articles. Titles and abstracts were preliminarily screened before the full texts were downloaded. Then we read the full texts to select eligible studies that meet the inclusion criteria. If there was any dissent on a study, a third researcher (Lei Chi) was consulted to assist in determining whether to include it. Before data extraction, a sheet of standard data extraction was prepared, including data source, Modified Rankin Scale cut-off value that was defined as a poor outcome, outcome prediction time, missing data processing methods, sample sizes of the training and validation sets, validation set confirmation way, internal and external validation information, predictors and their number, as well as ML model types. Data of the accuracy metrics were also collected, including sensitivity, specificity, receiver operator characteristic (ROC), area under the curve (AUC) and other.

### 2.4. Quality analysis

The ROB of included studies was assessed using PROBAST (18). It involves four major domains: participants, predictors, outcomes and statistic analysis, and reflects the overall ROB and applicability. The four domains include two, three, six and nine signaling questions, respectively. Signaling questions are answered as yes/probable yes (Y/PY), no/probably no (N/PN), or no information (NI). If a domain is answered with at least a N/PN, it is considered at high ROB. When all of the four domains were rated as low ROB, the overall ROB is deemed to be low. Two researchers (CJ and KZ) independently carried out ROB assessment in accordance with PROBAST. Then their assessment results were cross checked. Any dissent was consulted to a third researcher (LW) for final determination.

### 2.5. Data analysis

We performed a meta-analysis of the metrics (C-statistics and accuracy) for evaluating ML models. If C-statistic lacked 95% confidence interval (CI) and standard error (SE), we referred to the study by Debray TP et al. (19) to estimate its standard error. In case of inaccurate original data, we calculated based on sensitivity and specificity in combination with the sample size of each molecular subtype and model. Given the difference in variables and parameters in ML models, a random-effects model was preferred to perform the meta-analysis. This meta-analysis was conducted using R4.2.0 (R development Core Team, Vienna, http://www.R-project.org). A subgroup analysis by ML models and Modified Rankin Scale threshold was performed in our systematic review and meta-analysis. Heterogeneity was quantified by calculating *I*^2^ as a percentage. A low level of heterogeneity was present when *I*^2^ was 25%, a moderate level when *I*^2^ was 50% and a high level when *I*^2^ was 75% (20). Publication bias was examined by creating a funnel plot and Begg's bias test.

## 3. Results

### 3.1. Literature search

**Figure 1:**
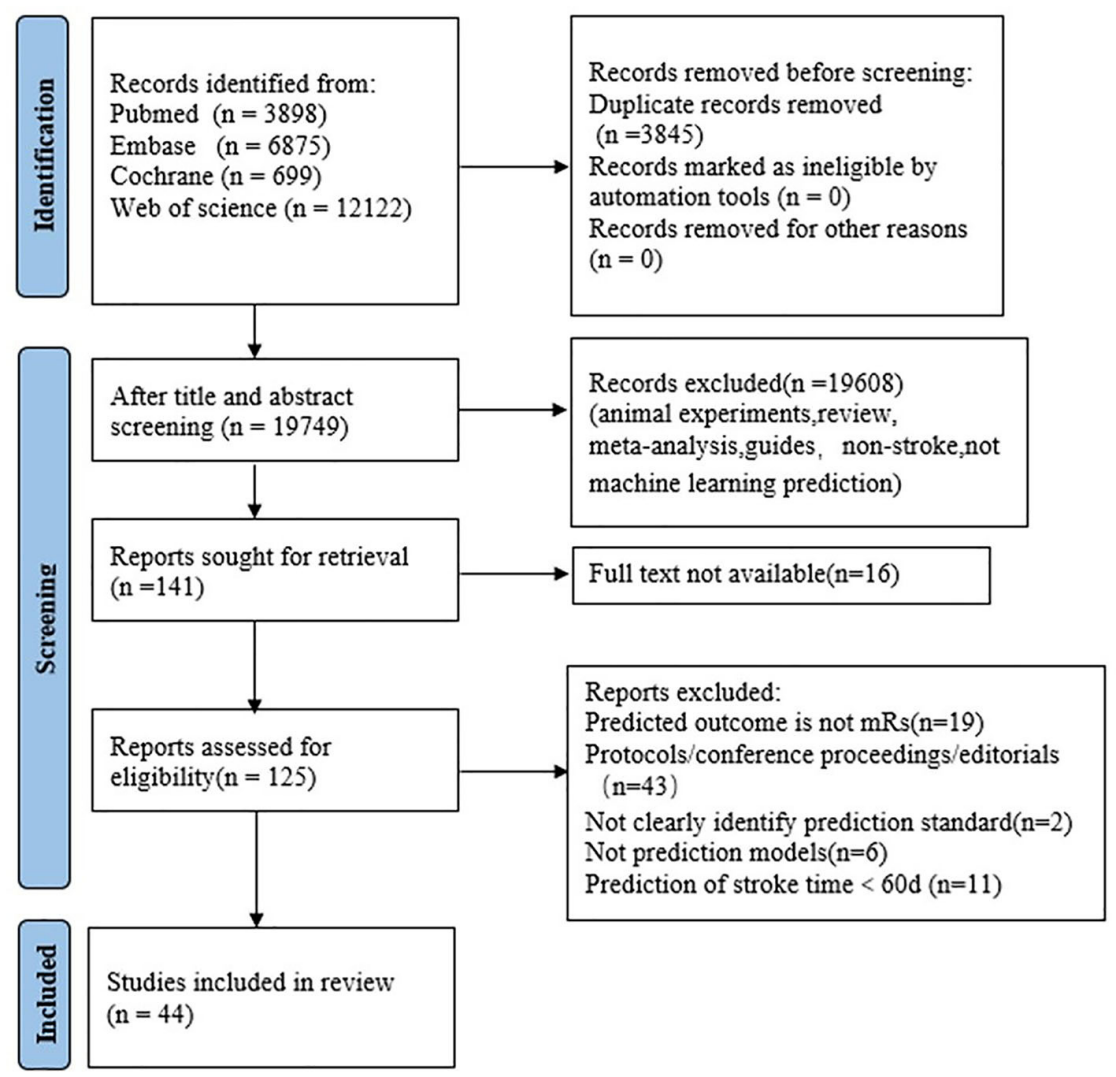
PRISMA flow diagram of study selection.

A total of 23,594 articles were initially searched from PubMed, Embase, Cochorane and Web of Science. In the screening process, not Modified Rankin Scale outcome, Modified Rankin Scale outcome time <90 days, not clearly identify prediction standard all exclusion conditions. After screening, a total of 44 papers were eligible (21–64). The selection process is shown in Figure 1.

### 3.2. Characteristics of included studies

A total of 44 eligible studies were included in this systematic review, involving 72,368 patients and 136 prediction models. All prediction models were internally validated, and 26 of them were externally validated. Additionally, among the 28 eligible studies, 15 studies were multi-centered (27–29, 39, 45, 47, 52, 55, 58–64); five studies extracted their original data from databases (24, 25, 32, 37, 57); and the remaining 24 studies were single-centered. Most eligible studies focused on IS and 7 studies (23, 32, 39, 40, 48, 60, 63) on HS. A study (32) used the same four models to predict the motor function in patients with IS and HS respectively. The primary outcome was Modified Rankin Scale at 3 to 6 months after the onset of stroke. Due to distinct purposes, these included studies defined poor motor function in a different manner. Specifically, it was defined as Modified Rankin Scale >1 in 4 studies (26, 31, 45, 58), Modified Rankin Scale >3 in 4 (39, 53, 60, 63) studies, Modified Rankin Scale >4 in two (25, 42) studies, and Modified Rankin Scale >2 in other 34 studies. In this study, a total of 13 primary studies using radiomics as predictive factors were identified (27, 30, 36, 37, 49, 51, 52, 54–57, 61, 62), from which 33 prediction models were extracted. Among them, 20 models primarily used MRI images as predictive factors.

In the training set, models of LR (Logistic Regression), RF (Random Forest), SVM (Support Vector Machine), XGB (Extreme Gradient Boosting), ANN (Artificial Neural Networks), DT (Decision Tree), GBM (Gradient Boosting Machine), DNN (Deep Neural Network), ADB (Adaptive Boosting), KNN (K-nearest Neighbors), CNN (Convolutional Neural Network) were applied. C-statistic was used for analysis. Meanwhile, the sensitivity and specificity of LR, RF, SVM, XGB, ANN, DT, ADB, CNN, Naive Bayes were calculated. In contrast, the validation set applied LR, ANN, RF, SVM, XGB, DT, GBM, KNN and GLM and used C-statistics to evaluate these models. The sensitivity and specificity of LR, RF, SVM, XGB, ANN, DT, and KNN were also calculated. Furthermore, these included studies were published from 2017 to 2023, which generally shows an increasing trend year by year. This reflects the rising popularity of ML prediction. Characteristics of included studies are presented in the Supplementary Tables S2–S4.

### 3.3. Quality assessment

**Figure 2:**
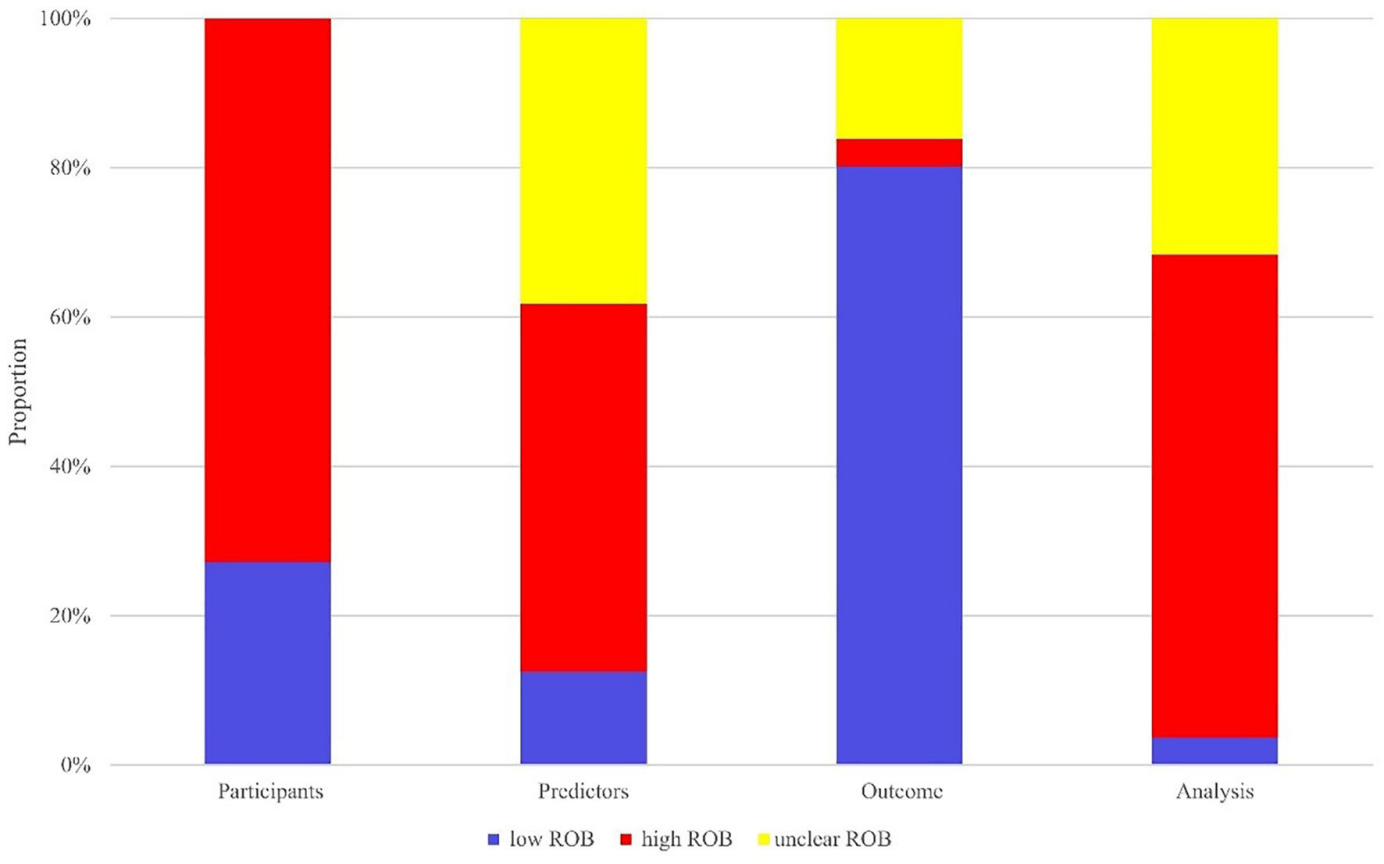
ROB evaluation.

In terms of the evaluation of case source, 99 of 136 models came from retrospective case-control studies, which was rated as high ROB. The high bias of data in retrospective studies caused limited accuracy of models in prediction. Thus these models were at high ROB. The other 37 models extracted clinical data from prospective cohort studies or registration data platforms, thereby rated at low ROB. Regarding the assessment of predictors, 67 models were rated at high ROB. Since researchers knew both predictors and data results in retrospective studies, there was a high ROB in such studies. For the outcome, 109 models were rated at low ROB, and 22 models were at unclear ROB for failing to report whether the predictor information was unclear at the time of outcome determination. Lastly, as for the analysis, 88 models were at high ROB, among them 60 models were rated at high ROB because of sample size <100 or events per variable(EPV) <10, and 43 models at unclear ROB due to a failure to elucidate the processing method of missing data, data complexity and optimal fitting method. The ROB evaluation is presented in Figure 2.

### 3.4. Variable ordering

**Figure 3:**
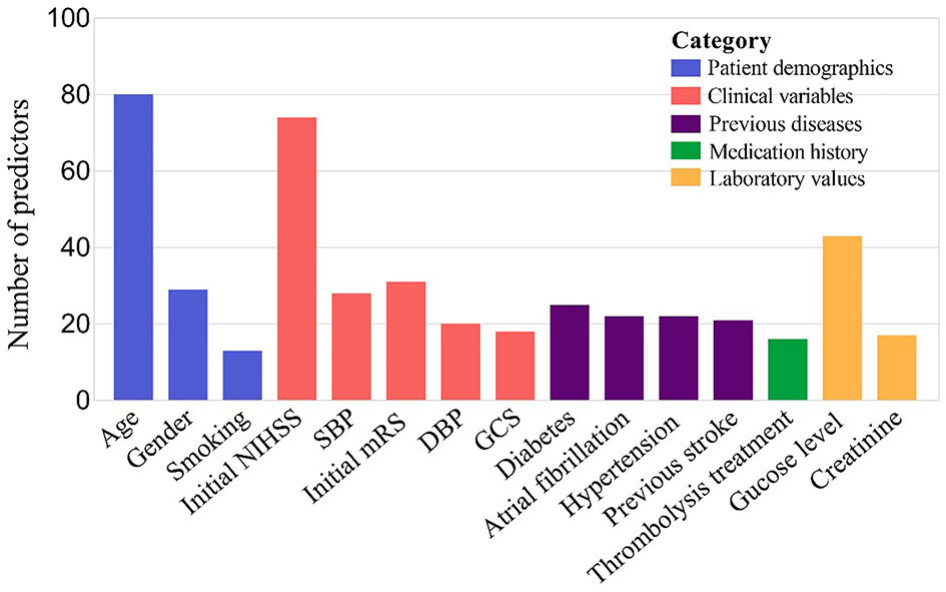
TOP 15 predictors 136 prognostic models for long-term post-stroke ML models. SBP, systolic blood pressure; DBP, diastolic blood pressure; GCS, Glasgow Coma Scale.

The present study summarized and ordered the predictors in included models. All predictors were divided into five categories, which comprehensively covered all-round information of stroke patients. This was helpful for clinicians to provide targeted secondary prevention and health guidance for corresponding patients in the future. Among all predictors, age (patient demographics) was used most frequently in prediction models, followed by initial NIHSS (clinical variables), glucose level (laboratory values), initial Modified Rankin Scale (clinical variables). In terms of the medication history, the frequency of thrombolysis treatment was high (shown in Figure 3).

### 3.5. Machine learning outcomes

**Figure 4:**
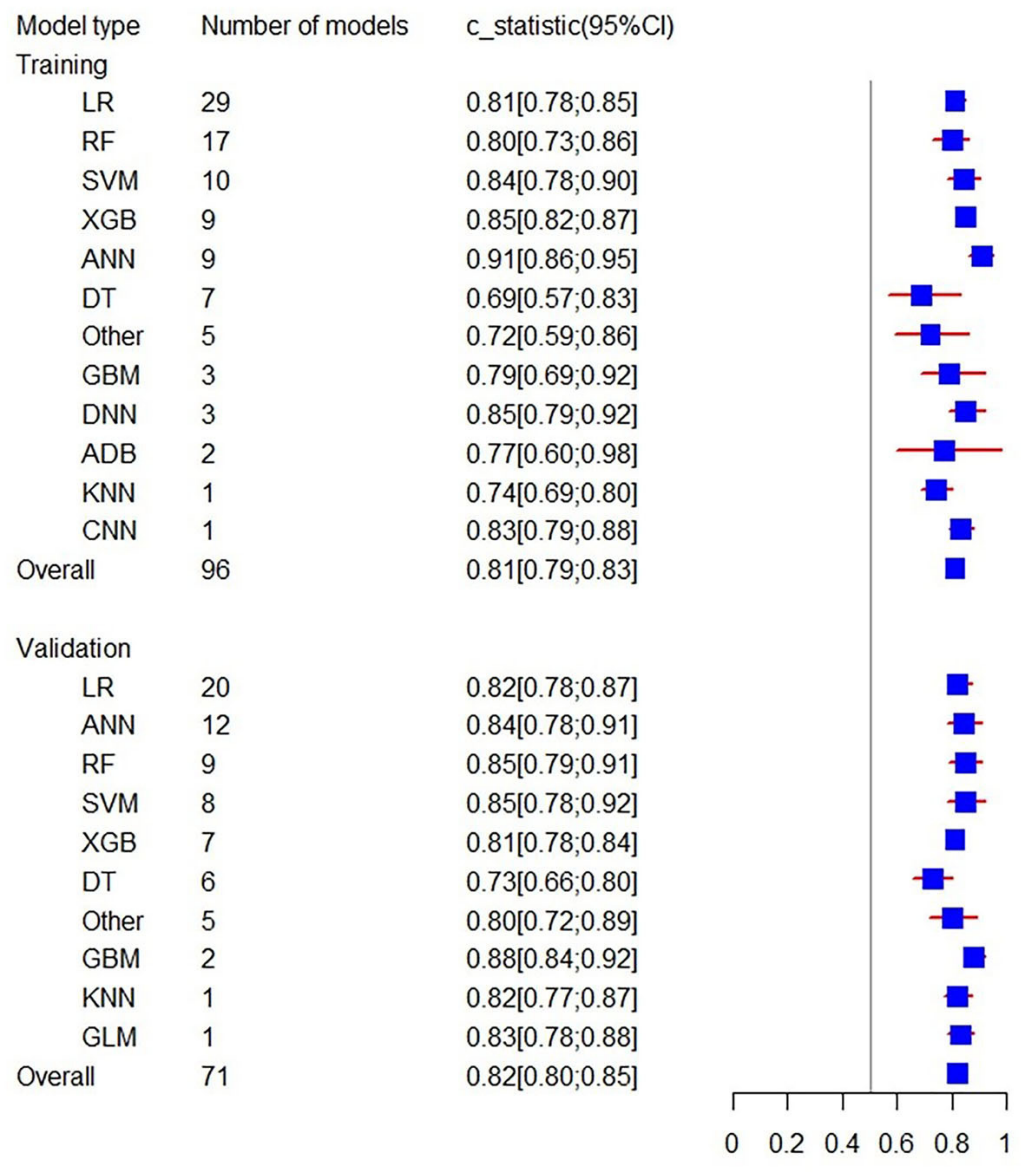
Forest plot of the overall C-statistics predicting 3–6 m Modified Rankin Scale outcomes in stroke patients.

Firstly, a random-effects model was used to combine the C-statistics in ML models. The overall C-statistic for the 96 models in the training set was 0.81 (95% CI: 0.79; 0.83). The 96 models can be classified into 12 types, which were ordered according to their frequency of application to prediction. Among the 12 model types, the LR model was used 29 times in all eligible studies to predict post-stroke motor function, and the C-statistic was 0.81 (95% CI: 0.78; 0.85). ANN had the best performance in prediction with a C-statistic of 0.91 (95% CI: 0.86; 0.95). The overall C-statistic for 71 models in the validation set was 0.82 (95% CI: 0.80; 0.85). The 71 models were divided into 10 types, which were also ranked according to their application frequency in prediction. Among the nine model types, LR models were used most frequently, namely 20 times, to predict post-stroke motor function, with a C-statistic of 0.82 (95% CI: 0.78; 0.87). The performance of each model can be seen in Table 1 and Figure 4.

**Table 1:** Overall C-statistics of machine learning models.

**Model**	**Training**			**Validation**		
	**Number of models**	**Sample size**	**C-statistics (95%CI)**	**Number of models**	**Sample size**	**C-statistics (95%CI)**
LR	29	17,459	0.81 [0.78; 0.85]	20	5,819	0.82 [0.78; 0.87]
RF	17	53,869	0.80 [0.73; 0.86]	9	43,629	0.85 [0.79; 0.91]
SVM	10	49,840	0.84 [0.78; 0.90]	8	43,466	0.85 [0.78; 0.92]
XGB	9	10,150	0.85 [0.82; 0.87]	7	3,138	0.81[0.78; 0.84]
ANN	9	86,701	0.91 [0.86; 0.95]	12	83,384	0.84 [0.78; 0.91]
DT	7	3,136	0.69 [0.57; 0.83]	6	3,342	0.73 [0.66; 0.80]
Other	5	2,972	0.72[0.59; 0.86]	5	1,115	0.80 [0.72; 0.89]
GBM	3	1,778	0.79 [0.69; 0.92]	2	326	0.88 [0.84; 0.92]
DNN	3	5,516	0.85 [0.79; 0.92]	NA	NA	NA
ADB	2	1,113	0.77 [0.60; 0.98]	NA	NA	NA
KNN	1	293	0.74	1	297	0.82
CNN	1	322	0.83	NA	NA	NA
GLM	NA	NA	NA	1	251	0.83
Overall	96	23,3149	0.81 [0.79; 0.83]	71	18,4767	0.82 [0.80; 0.85]

**Figure 5:**
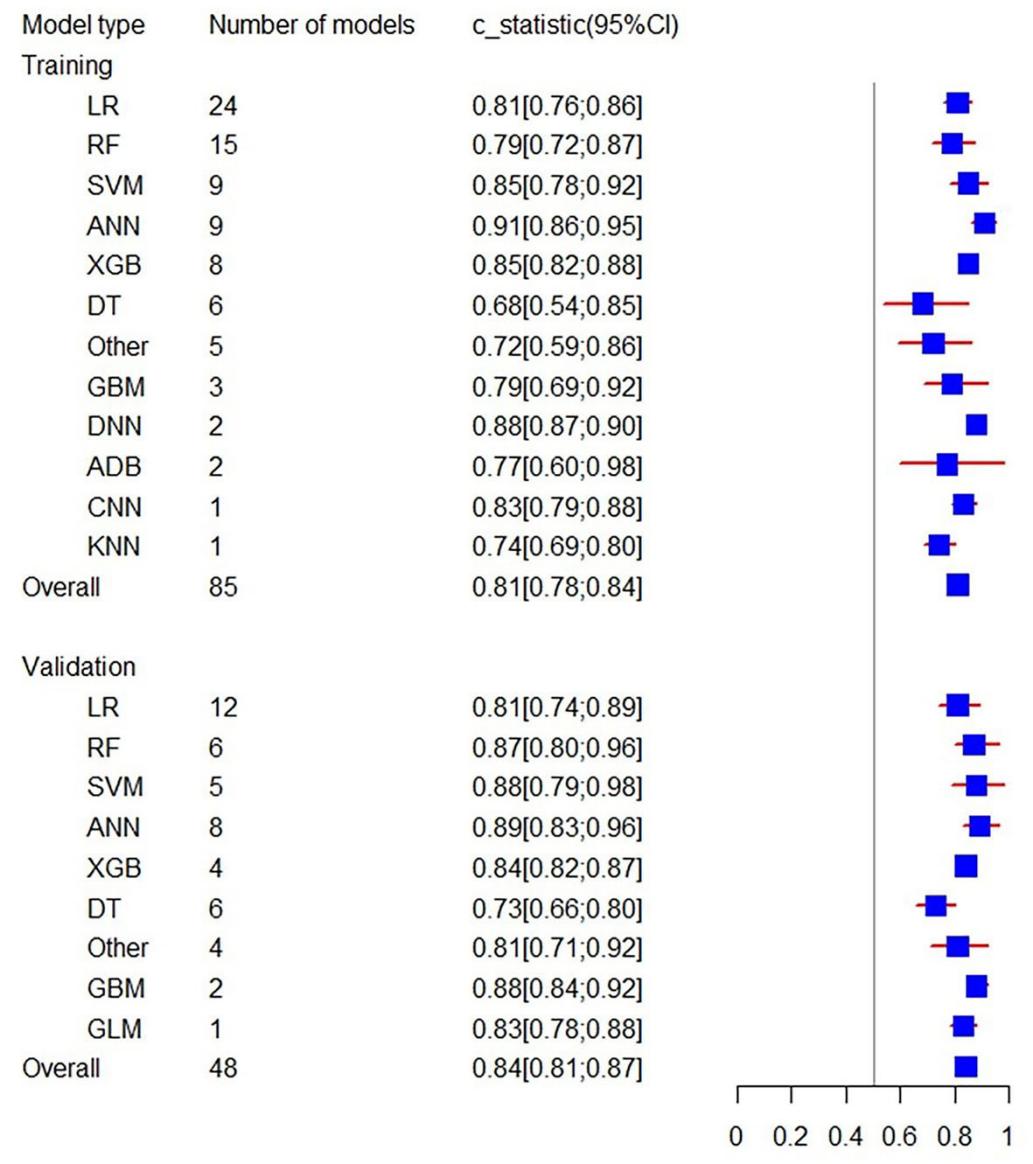
Forest plot of the C-statistics predicting 3–6 m Modified Rankin Scale >2 outcomes in stroke patients.

To minimize the heterogeneity of data sources, the present study also performed C-statistic pooling based on different Modified Rankin Scale cutoff thresholds. Among the training set models with Modified Rankin Scale >2, there were 85 in total with a C-statistic of 0.81 (95% CI: 0.78; 0.84), and among the validation set models, there were 48 in total with a C-statistic of 0.84 (95% CI: 0.81; 0.87). In both the prediction model of training and validation sets, the ANN prediction performed the best with C-statistics of 0.91 (95% CI: 0.86; 0.95) and 0.89 (95% CI: 0.83; 0.96), respectively, as shown in Figure 5. For models with Modified Rankin Scale > 1, there were 6 training set models, with an overall C-statistic of 0.78 (95% CI: 0.77; 0.80). There were 14 validation set models, with an overall C-statistic of 0.79 (95% CI: 0.77; 0.82). For models with Modified Rankin Scale >3, there were 5 training set models, with an overall C-statistic of 0.83 (95% CI: 0.78; 0.88). There were 4 validation set models, all of which were LR models, with an overall C-statistic of 0.87 (95% CI: 0.82; 0.92). There were no training set models with Modified Rankin Scale > 4, but there were 5 validation set models, with an overall C-statistic of 0.79 (95% CI: 0.78; 0.81). The performance of each model within the subgroups can be seen in Table 2.

**Table 2:** C-statistics of subgroups machine learning models (according to threshold of Modified Rankin Scale).

**Subgroup**	**Training**			**Validation**		
	**Number of models**	**Sample size**	**C-statistics (95%CI)**	**Number of models**	**Sample size**	**C-statistics (95%CI)**
**Modified Rankin Scale** >**1**						
LR	2	2,012	0.76 [0.74; 0.79]	3	1,148	0.82 [0.76; 0.88]
XGB	1	1,524	0.81	2	678	0.78 [0.72; 0.84]
RF	1	1,524	0.78	2	678	0.79 [0.73; 0.86]
DNN	1	1,524	0.78	NA	NA	NA
SVM	1	1,524	0.77	2	678	0.81 [0.73; 0.90]
KNN	NA	NA	NA	1	297	0.82
ANN	NA	NA	NA	3	443	0.66 [0.53; 0.82]
Other	NA	NA	NA	1	31	0.75
Overall	6	8,108	0.78 [0.77; 0.80]	14	3,953	0.79 [0.77; 0.82]
**Modified Rankin Scale** >**2**						
LR	24	13,742	0.81 [0.76; 0.86]	12	2,578	0.81 [0.74; 0.89]
RF	15	52,067	0.79 [0.72; 0.87]	6	41,425	0.87 [0.80; 0.96]
SVM	9	48,316	0.85 [0.78; 0.92]	5	41,262	0.88 [0.79; 0.98]
ANN	9	86,701	0.91 [0.86; 0.95]	8	81,415	0.89 [0.83; 0.96]
XGB	8	8,626	0.85 [0.82; 0.88]	4	934	0.84 [0.82; 0.87]
DT	6	2,858	0.68 [0.54; 0.85]	6	3,342	0.73 [0.66; 0.80]
Other	5	2,972	0.72 [0.59; 0.86]	4	1,084	0.81 [0.71; 0.92]
GBM	3	1,778	0.79 [0.69; 0.92]	2	326	0.88 [0.84; 0.92]
DNN	2	3,992	0.88 [0.87; 0.90]	NA	NA	NA
ADB	2	1,113	0.77 [0.60; 0.98]	NA	NA	NA
CNN	1	322	0.83	NA	NA	NA
KNN	1	293	0.74	NA	NA	NA
GLM	NA	NA	NA	1	251	0.83
Overall	85	222,780	0.81 [0.78; 0.84]	48	172,617	0.84 [0.81; 0.87]
**Modified Rankin Scale** >**3**						
LR	3	1,705	0.86 [0.84; 0.89]	4	567	0.87 [0.82; 0.92]
RF	1	278	0.82	NA	NA	NA
DT	1	278	0.75	NA	NA	NA
Overall	5	2,261	0.83 [0.78; 0.88]	4	567	0.87 [0.82; 0.92]
**Modified Rankin Scale** >**4**						
ANN	NA	NA	NA	1	1,526	0.81
LR	NA	NA	NA	1	1,526	0.80
RF	NA	NA	NA	1	1,526	0.80
XGB	NA	NA	NA	1	1,526	0.78
SVM	NA	NA	NA	1	1,526	0.77
Overall	NA	NA	NA	5	7,630	0.79 [0.78; 0.81]

**Figure 6:**
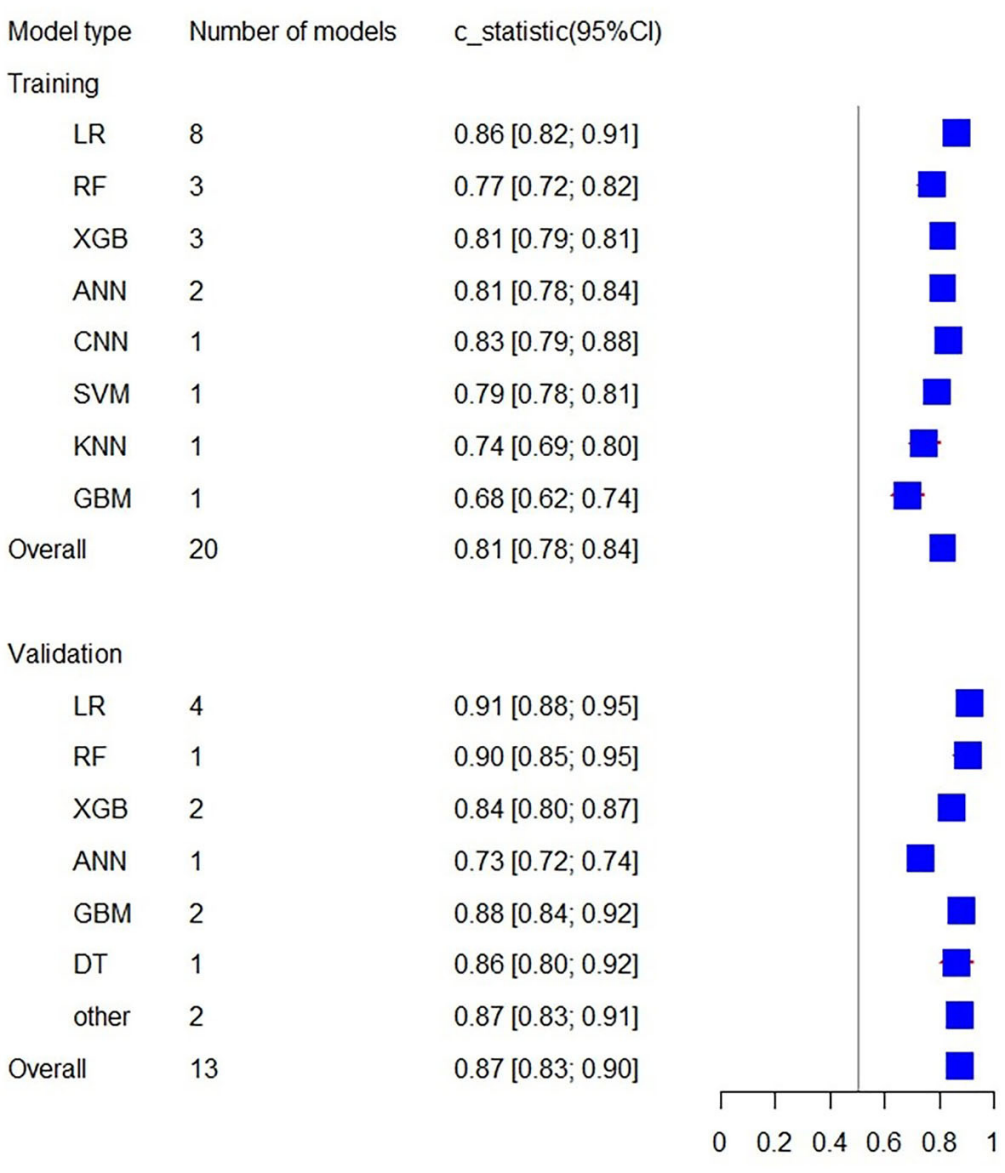
Forest plot of the C-statistics predicting 3–6 m Modified Rankin Scale outcomes in stroke patients (radiomics-based predictors).

Additionally, the C-statistics were also combined for radiomics-based machine learning prediction models. The training set included a total of 20 prediction models from 8 categories. The overall C-statistic was 0.81 (95% CI: 0.78; 0.84). The most numerous type was the LR model, which also had the best predictive performance with a C-statistic of 0.86 (95% CI: 0.82; 0.91). There were 13 validation set models, with an overall C-statistic of 0.87 (95% CI: 0.83; 0.90). Similarly, the LR model had the best predictive performance with a C-statistic of 0.91 (95% CI: 0.88; 0.95). The performance of the remaining models is provided in Table 3 and Figure 6.

**Table 3:** C-statistics of radiomics-based predictors machine learning models.

**Model**	**Training**			**Validation**		
	**Number of models**	**Sample size**	**C-statistics (95%CI)**	**Number of models**	**Sample size**	**C-statistics (95%CI)**
LR	8	4,055	0.86 [0.82; 0.91]	4	350	0.91 [0.88; 0.95]
RF	3	4,595	0.77 [0.72; 0.82]	1	163	0.90
XGB	3	4,725	0.81 [0.79; 0.81]	2	456	0.84 [0.80; 0.87]
ANN	2	3,251	0.81 [0.78; 0.84]	1	74	0.73
CNN	1	322	0.83	NA	NA	NA
SVM	1	3,001	0.79	NA	NA	NA
KNN	1	293	0.74	NA	NA	NA
GBM	1	293	0.68	2	326	0.88 [0.84; 0.92]
DT	NA	NA	NA	1	163	0.86
Other	NA	NA	NA	2	326	0.87 [0.83; 0.91]
Overall	20	20,535	0.81 [0.78; 0.84]	13	1858	0.87 [0.83; 0.90]

### 3.6. Sensitivity and specificity

**Figure 7:**
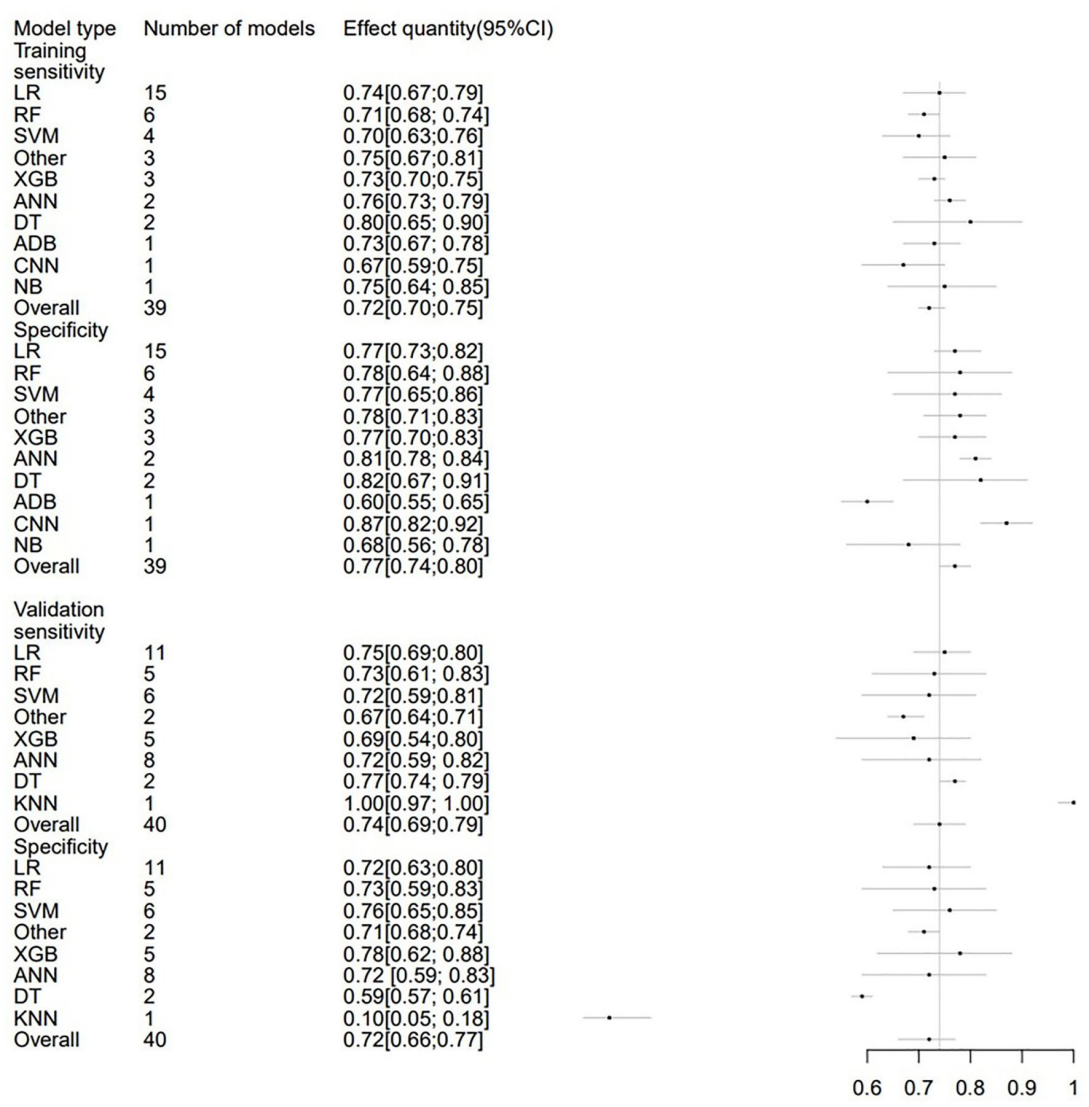
Forest plot of the sensitivity and specificity predicting 3–6 m Modified Rankin Scale outcomes in stroke patients.

In order to avoid data imbalance, the sensitivity and specificity of models in prediction were analyzed. The training set included 39 models in total with the overall sensitivity of 0.72 (95% CI: 0.70; 0.75). LR were used 15 times, with a sensitivity of 0.74 (95% CI: 0.67; 0.79). Additionally, the overall specificity was 0.77 (95% CI: 0.74; 0.80). The validation set included 40 models, and the overall sensitivity and specificity were 0.74 (95%CI: 0.69; 0.79) and 0.72 (95% CI: 0.66; 0.77), respectively. Sensitivity and specificity analyses were also conducted for subgroups with different Modified Rankin Scale thresholds. The sensitivity and specificity of ML models and subgroups were presented in Table 4 and Figure 7.

**Table 4:** Sensitivity and specificity of overall and subgroup machine learning models.

**Subgroup**	**Training**				**Validation**			
	**Number of models**	**Sample size**	**Sen (95%CI)**	**Spe (95%CI)**	**Number of models**	**Sample size**	**Sen (95%CI)**	**Spe (95%CI)**
**Model type**								
LR	15	10,637	0.74 [0.67;0.79]	0.77 [0.73;0.82]	11	4,204	0.75 [0.69;0.80]	0.72 [0.63;0.80]
RF	6	2,598	0.71 [0.68; 0.74]	0.78 [0.64; 0.88]	5	3,058	0.73 [0.61; 0.83]	0.73 [0.59; 0.83]
SVM	4	4,592	0.70 [0.63;0.76]	0.77 [0.65;0.86]	6	3,189	0.72 [0.59;0.81]	0.76 [0.65;0.85]
Other	3	387	0.75 [0.67;0.81]	0.78 [0.71;0.83]	2	538	0.67 [0.64;0.71]	0.71 [0.68;0.74]
XGB	3	5,022	0.73 [0.70;0.75]	0.77 [0.70;0.83]	5	2,682	0.69 [0.54; 0.80]	0.78 [0.62; 0.88]
ANN	3	3,513	0.76 [0.73; 0.79]	0.81 [0.78; 0.84]	8	2,720	0.72 [0.59; 0.82]	0.72 [0.59; 0.83]
DT	2	724	0.80 [0.65; 0.90]	0.82 [0.67; 0.91]	2	1,014	0.77 [0.74; 0.79]	0.59 [0.57; 0.61]
ADB	1	614	0.73	0.60	NA	NA	NA	NA
CNN	1	322	0.67	0.87	NA	NA	NA	NA
NB	1	150	0.75	0.68	NA	NA	NA	NA
KNN	0	NA	NA	NA	1	297	1.00	0.10
**Modified Rankin Scale**								
>1	0	NA	NA	NA	13	3,483	0.85 [0.76;0.91]	0.57 [0.46;0.68]
>2	38	27,724	0.72 [0.69;0.75]	0.77 [0.74;0.80]	20	6,302	0.68 [0.62;0.72]	0.79 [0.72;0.84]
>3	1	835	0.75	0.70	2	287	0.84 [0.75;0.90]	0.68 [0.63;0.72]
>4	0	NA	NA	NA	5	7,630	0.65 [0.56;0.73]	0.80 [0.72;0.86]
Overall	39	28,559	0.72 [0.70;0.75]	0.77 [0.74;0.80]	40	17,702	0.74 [0.69;0.79]	0.72 [0.66;0.77]

### 3.7. Publication bias

**Figure 8:**
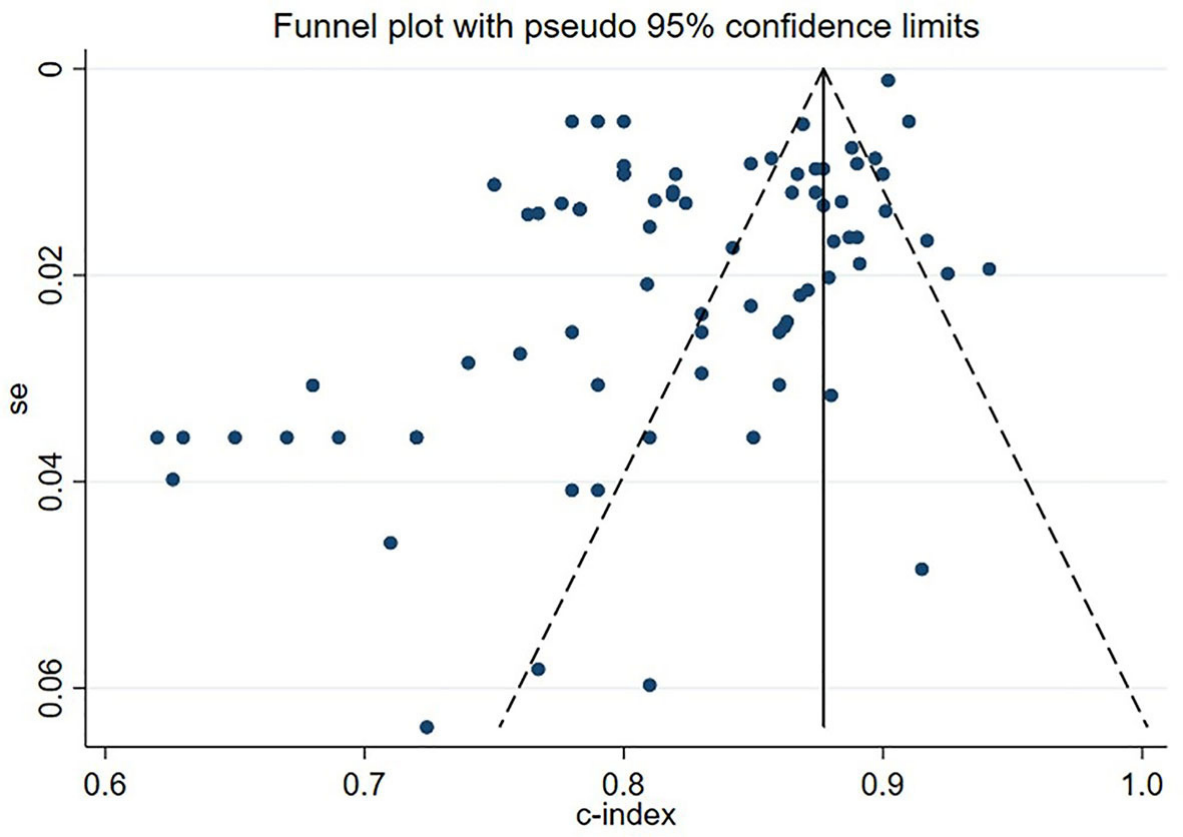
Funnel plot for publication bias of C-statistics in the training set.

**Figure 9:**
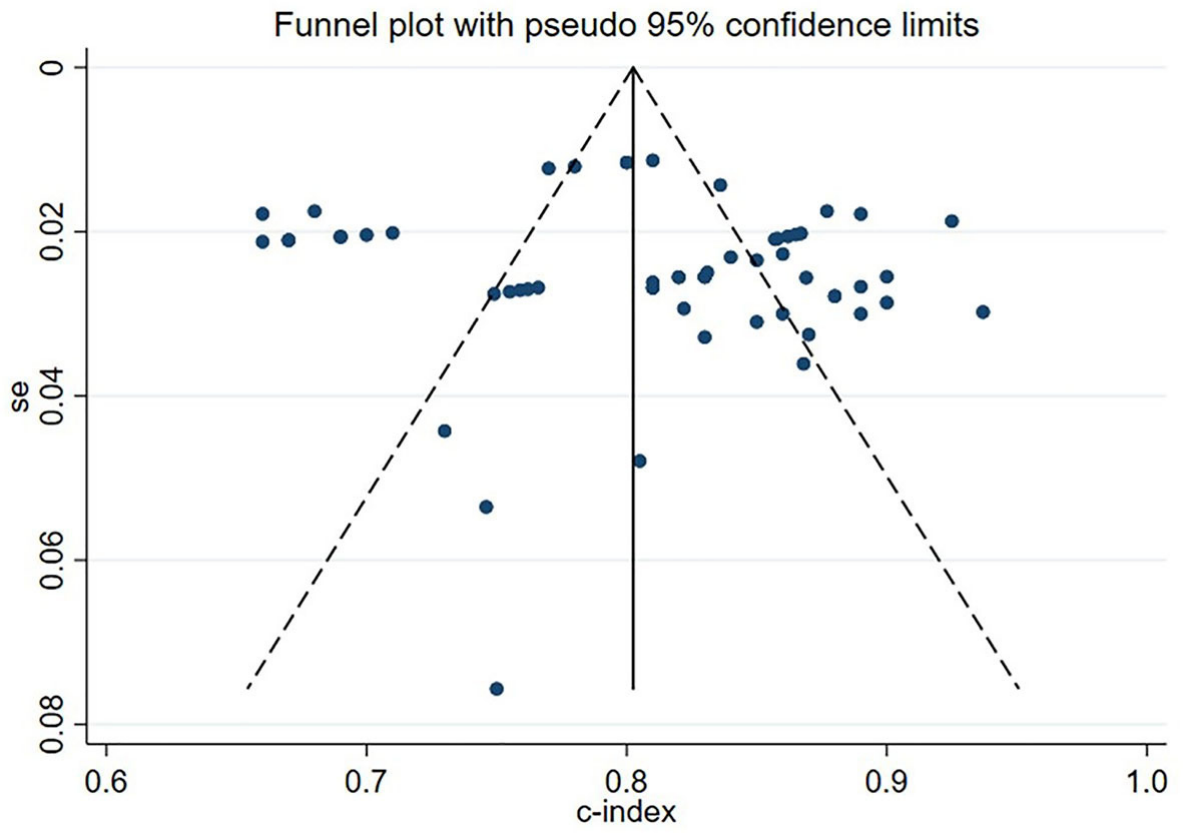
Funnel plot for publication bias of C-statistics in the validation set.

**Figure 10:**
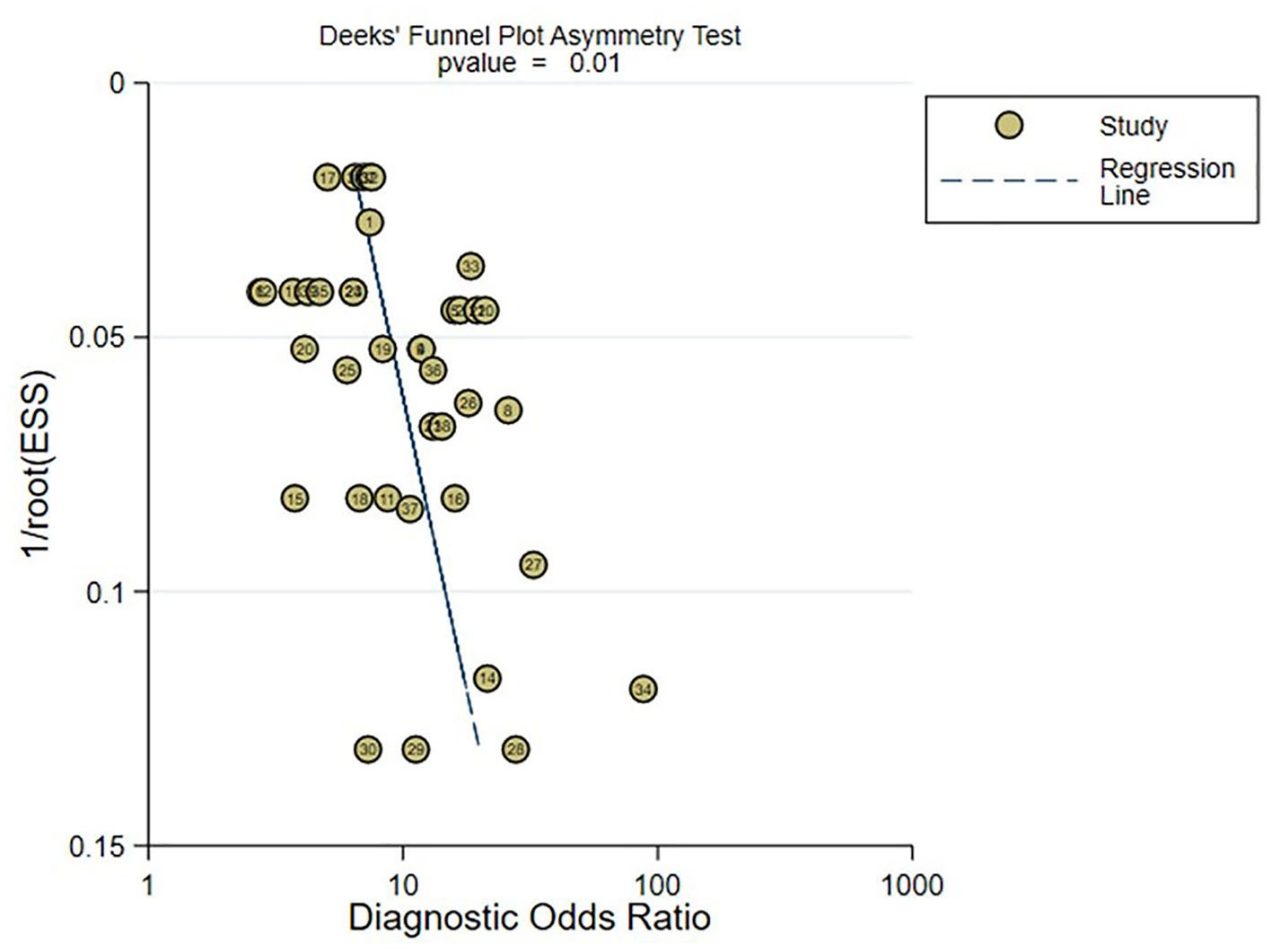
Funnel plot for publication bias of diagnostic 4-fold table in the training set.

**Figure 11:**
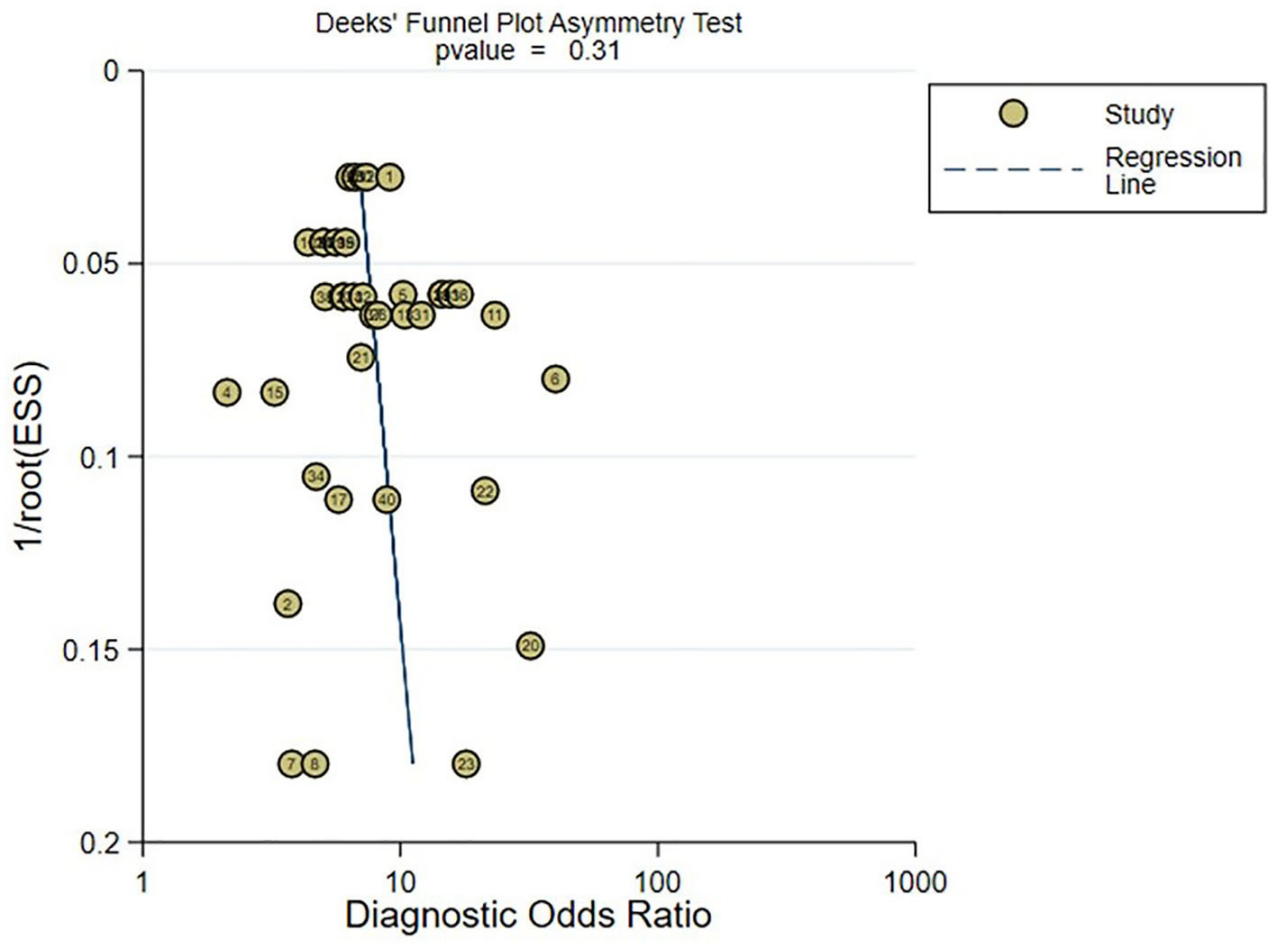
Funnel plot for publication bias of diagnostic 4-fold table in the validation set.

In the meta-analysis of C-statistic, no publication bias was found in the funnel plots for both the training set and validation set of ML models for predicting motor function 3–6 months after stroke. The results of Begg test showed that *P* = 0.473 in the training set, P = 0.909 in the verification set. The funnel plots are shown in Figures 8, 9. In the meta-analysis of the diagnostic 4-fold table, there was publication bias in the training set of ML models for predicting motor function 3–6 months after stroke, while no publication bias was found in the validation set. The Beg test results showed that *P* = 0.01 in the training set, *P* = 0.31 in the validation set. The funnel plots are shown in Figures 10, 11.

## 4. Discussion

The study reviews the performance of ML models in the prediction of motor function recovery in patients 3–6 months post-stroke. In the case of Modified Rankin Scale >1, >2, >3, >4, the model's predictive performance was favorable. The C-statistics for models with predictive factors based on radiomics were 0.81 (95% CI: 0.78; 0.84) in the training set and 0.87 (95% CI: 0.83; 0.90) in the validation set. The overall sensitivity and specificity of the models were both over 0.70 and relatively balanced. The study makes up the gap and deficiency in current researches on the prediction of motor function recovery in stroke patients, which has significant instruction for clinical practice. In the quality assessment part, the ROB of original studies was analyzed in accordance with the PROBAST standard. This provides detailed suggestions for researchers to design model prediction tests in the future, which is conducive to standardization and unification.

According to this analysis of predictors, age and initial NIHSS, are the most critical predictors for the prognosis of motor function in patients with stroke for 90 to 180 days, followed by glucose level, initial Modified Rankin Scale. This is consistent with the results of previous studies (2, 65, 66) on prediction models and meta-analysis of predictors. A large number of studies show that aged patients with acute IS have higher mortality and poorer quality of life than their young counterparts. For instance, both cerebral infarction complicated with pulmonary infection and hemorrhagic transformation after cerebral infarction are more likely to cause relatively poor outcomes in aged patients (67, 68). NIHSS score has been widely recognized as a key determinant of the prognosis in patients with acute IS in China and abroad (69). Furthermore, a previous study (70) on motor function outcome in stroke patients show that initial measures were found to be the most significant predictors of upper limb recovery; odds ratio 14.84 (95% CI 9.08–24.25) and 38.62 (95% CI 8.40–177.53) respectively.

AI has been widely applied in the diagnosis, classification, and prediction of stroke. One of its biggest advantages is that it can process data endlessly and can perform faster than traditional computer-aided detection and diagnosis (CAD) (71). Although there are more and more studies on post-stroke motor function prognosis, there is still a lack of prediction and guidance for neurological motor function, especially for model evaluation after stratifying prediction outcomes. A previous systematic review (72) collected and summarized clinical prognosis trials for patients with large vessel occlusion undergoing thrombectomy, and predicted 90-day Modified Rankin Scale for 802 patients. The random-effects model showed an AUC of 0.846 (95% CI 0.686–0.902), indicating good predictive performance. However, as most of the limited number of included studies used SVM, a comprehensive comparison of the predictive performance of various models to determine the best model was not possible. Similarly, earlier research (73) in rehabilitation medicine on the prediction of post-stroke function recovery confirmed the application of ML prediction ability in clinical settings but did not provide specific C-statistic values, making it difficult to accurately assess predictive performance. Therefore, conducting a meta-analysis of ML motor function prediction models classified by prognosis outcome and predictive factors is both necessary and valuable, as it deepens the understanding of earlier research and further clarifies the ideal application value of ML in predicting post-stroke outcomes.

For stroke prediction, most existing ML algorithms use binary classification to evaluate the outcome indicator. Conventionally, when Modified Rankin Scale are 0–2, functional outcomes are usually defined as “good”; when Modified Rankin Scale 3–6, functional outcomes are typically defined as “poor.” Studies usually measure Modified Rankin Scale at 90 days post-stroke (38, 74, 75). However, with different patient situations in clinical settings and various research objectives, studies have started using Modified Rankin Scale threshold values such as 0–1 VS 2–6, 0–3 VS 4–6, and conducting later follow-ups. New ML algorithms that incorporate these results will provide greater assistance to clinicians.

The advancement of machine learning has made it possible to transform subjective visual interpretation into objective evaluation driven by image data. Radiomics has emerged in this context. Radiomics is a computer-aided process that can extract a large number of quantitative features from biomedical images in an objective, repeatable, and high-throughput manner (76, 77). These features can be combined with other medical information such as demographics, clinical, histological, or genomic data to improve clinical treatment decision-making and accelerate the progress of precision medicine. A systematic review (78) reveals that the artificial intelligence coupling CNN with image feature has greater sensitivity, up to 83%. ML not only offers promising applications in medical imaging by learning information features and patterns from structured input data, but also promotes the emergence of deep learning (DL) and demonstrates its excellent performance in medical image processing (79, 80). XinruiWang's latest study (81) analyzed ML models to predict the volume of core infarct tissue in AIS patients based on basic CT or MRI imaging at admission. DL models outperformed traditional ML classifiers, with the best performance observed in DL algorithms combined with CT data. Currently, the pooled dice similarity coefficient score of the included ML models for final infarct prediction based on ML was 0.50 (95% CI 0.39–0.61).

In theory, different imaging modalities and parameters provide different diagnostic and prognostic information that can complement each other. Therefore, adjusting and optimizing parameters for multimodal imaging data in radiomics can improve the overall predictive performance. In addition, the study combined clinical data such as clinical symptom assessment, medical history, and laboratory examinations (82, 83). Multidimensional input information consisting of both imaging and clinical data has the potential to establish better prediction models, which is a direction for future research.

### 4.1. Limitation

However, there are some limitations to the present study that need to be considered. First, due to the different types of algorithms and parameter adjustments, there is inevitably a high degree of heterogeneity between studies. To minimize the heterogeneity, we conducted subgroup analysis according to different Modified Rankin Scale cut-off values and analyzed the performance of predictive factors based on different categories. Moreover, from the summary plot of variables, it can be observed that the predictive factors in each study are similar to some degree and selected from five dimensions. Second, although ML has enormous potential in the computing function of huge data, its “black box” characteristic restricts clinicians from trusting the ML prediction. Meanwhile, due to the instability of association between impact factors, ML model requires plenty of samples to improve its accuracy (84, 85). Third, while using the Modified Rankin Scale as a functional outcome measure can directly elucidate the functional levels, it fails to express the details of various post-stroke neurological symptoms, such as dysarthria and pure sensory stroke. At last, from the literature quality assessment summary table, retrospective case studies are in the majority, leading to a high ROB and poor performance of prediction models. Therefore, in order to avoid high ROB, future clinical studies on ML prediction should collect data from clinical registration platforms or prospective clinical studies. Additionally, the design of clinical protocol should meet the requirement of EPV ≥20 to ensure the reliability of the results of the prediction model.

## 5. Conclusion

In this study, we conducted a systematic review and meta-analysis of the current research using ML algorithms to predict post-stroke motor function 3–6 months. Due to its good predictive performance, sensitivity, and specificity, ML can be used as an evaluation tool for predicting motor function after stroke. Additionally, the study found that ML models with radiomics as predictive variables also demonstrated good predictive capabilities. The multidimensional input information consisting of both imaging and clinical data has the potential to establish better prediction models that can guide clinical work.

## Data availability statement

The original contributions presented in the study are included in the article/Supplementary material, further inquiries can be directed to the corresponding author.

## Author contributions

QL wrote the main manuscript and fully participated in all analyses. XL, LC, and LW contributed to the study concept and design. WZ, CJ, XZ, and KZ participated in literature search, data extraction, and quality assessment. All authors have read and approved the final manuscript.
