# Supplementary material for: Machine learning prediction of motor function in chronic stroke patients: a systematic review and meta-analysis
Source: Front Neurol. 2023 Jun 13;14:1039794. doi: 10.3389/fneur.2023.1039794 (PMC10299899; doi:10.3389/fneur.2023.1039794)
Supplement: Supplementary file 1 [file Table_1.DOCX]

Table S1 Literature search strategy

**1.Pubmed**

| Search number | Query | Results |
| --- | --- | --- |
| #1 | "Stroke"[Mesh] | 169373 |
| #2 | ((((((((((Stroke[Title/Abstract]) OR (Strokes[Title/Abstract])) OR (Cerebrovascular Accident[Title/Abstract])) OR (Cerebrovascular Accidents[Title/Abstract])) OR (Cerebrovascular Apoplexy[Title/Abstract])) OR (Apoplexy, Cerebrovascular[Title/Abstract])) OR (Vascular Accident, Brain[Title/Abstract])) OR (Brain Vascular Accident[Title/Abstract])) OR (Brain Vascular Accidents[Title/Abstract])) OR (Vascular Accidents, Brain[Title/Abstract])) OR (Apoplexy[Title/Abstract]) | 320960 |
| #3 | #1 OR #2 | 358547 |
| #4 | "Machine Learning"[Mesh] | 55346 |
| #5 | (((((((((((((((machine learning[Title/Abstract]) OR (Transfer Learning[Title/Abstract])) OR (Deep learning[Title/Abstract])) OR (Prediction model[Title/Abstract])) OR (artificial intelligence[Title/Abstract])) OR (random forest[Title/Abstract])) OR (artificial neural network[Title/Abstract])) OR (ANN[Title/Abstract])) OR (Support vector machine[Title/Abstract])) OR (SVM[Title/Abstract])) OR (Gradient Boosting Machine[Title/Abstract])) OR (GBM[Title/Abstract])) OR (Nomogram[Title/Abstract])) OR (XGboost[Title/Abstract])) OR (Decision tree[Title/Abstract])) OR (External validation[Title/Abstract]) | 243790 |
| #6 | #4 OR #5 | 250078 |
| #7 | #3 AND #6 | 3898 |

**2.Cochrane**

| Search number | Query | Results |
| --- | --- | --- |
| #1 | MeSH descriptor: [Stroke] explode all trees | 12134 |
| #2 | (Stroke):ti,ab,kw OR (Strokes):ti,ab,kw OR (Cerebrovascular Accident):ti,ab,kw OR (Cerebrovascular Apoplexy):ti,ab,kw OR (Apoplexy, Cerebrovascular):ti,ab,kw | 68675 |
| #3 | (Vascular Accident, Brain):ti,ab,kw OR (Brain Vascular Accident):ti,ab,kw OR (Brain Vascular Accidents):ti,ab,kw OR (Vascular Accidents, Brain):ti,ab,kw OR (Apoplexy):ti,ab,kw | 1023 |
| #4 | #1 OR #2 OR #3 | 67058 |
| #5 | MeSH descriptor: [Machine Learning] explode all trees | 532 |
| #6 | (machine learning):ti,ab,kw OR (Transfer Learning):ti,ab,kw OR (Deep learning):ti,ab,kw OR (Prediction model):ti,ab,kw OR (artificial intelligence):ti,ab,kw | 9873 |
| #7 | (random forest):ti,ab,kw OR (artificial neural network):ti,ab,kw OR (ANN):ti,ab,kw OR (Support vector machine):ti,ab,kw OR (SVM):ti,ab,kw | 4002 |
| #8 | (Gradient Boosting Machine):ti,ab,kw OR (GBM):ti,ab,kw OR (Nomogram):ti,ab,kw OR (XGboost):ti,ab,kw OR (Decision tree):ti,ab,kw | 4305 |
| #9 | (External validation):ti,ab,kw | 1387 |
| #10 | #5 OR #6 OR #7 OR #8 OR #9 | 15309 |
| #11 | #4 AND #10 | 699 |

**3.Embase**

| Search number | Query | Results |
| --- | --- | --- |
| #1 | 'cerebrovascular accident'/exp | 395128 |
| #2 | stroke:ab,ti OR strokes:ab,ti OR 'cerebrovascular accident':ab,ti OR 'cerebrovascular accidents':ab,ti OR 'cerebrovascular apoplexy':ab,ti OR 'apoplexy, cerebrovascular':ab,ti OR 'vascular accident, brain':ab,ti OR 'brain vascular accident':ab,ti OR 'vascular accidents, brain':ab,ti OR apoplexy:ab,ti | 498439 |
| #3 | #1 OR #2 | 602353 |
| #4 | 'machine learning'/exp | 132708 |
| #5 | 'machine learning':ab,ti OR 'transfer learning':ab,ti OR 'deep learning':ab,ti OR 'prediction model':ab,ti OR 'artificial intelligence':ab,ti OR 'random forest':ab,ti OR 'artificial neural network':ab,ti OR ann:ab,ti OR 'support vector machine':ab,ti OR svm:ab,ti OR 'gradient boosting machine':ab,ti OR gbm:ab,ti OR nomogram:ab,ti OR xgboost:ab,ti OR 'decision tree':ab,ti OR 'external validation':ab,ti | 342345 |
| #6 | #4 OR #5 | 372875 |
| #7 | #3 AND #6 | 6875 |

**4.Web of science**

| Search number | Query | Results |
| --- | --- | --- |
| #1 | Stroke（Topic）or Strokes（Topic）or Cerebrovascular Accident （Topic） or CerebrovascularApoplexy（Topic）or Apoplexy，Cerebrovascular（Topic）or Vascular Accident，Brain （Topic） or Brain Vascular Accident （Topic） or Brain Vascular Accidents （Topic） or VascularAccidents，Brain（Topic）or Apoplexy（Topic） | 439159 |
| #2 | machine learning（Topic） or Transfer Learning （Topic） or Deep learning （Topic） orPrediction model（Topic） or artificial intelligence （Topic） or random forest（Topic）orartificial neural network （Topic） or ANN （Topic） or Support vector machine （Topic） orSVM（Topic） or Gradient Boosting Machine（Topic） or GBM （Topic） or Nomogram （Topic） orXGboost（Topic） or Decision tree（Topic）or External validation （Topic） | 1774255 |
| #3 | #1 AND #2 | 12122 |
