# Supplementary material for: Machine learning prediction of motor function in chronic stroke patients: a systematic review and meta-analysis
Source: Front Neurol. 2023 Jun 13;14:1039794. doi: 10.3389/fneur.2023.1039794 (PMC10299899; doi:10.3389/fneur.2023.1039794)
Supplement: Supplementary file 2 [file Table_2.DOCX]

Table S2 Patients basic information

| NO. | First author and year | Dataset  Source | Study type | Disease type | Total patient number (n) | Training  sets (n) | Test set (n) | External test set | follow-up duration | mRS cutoff value |
| --- | --- | --- | --- | --- | --- | --- | --- | --- | --- | --- |
| 1 | Esra Zihni  2020 | Single center | retrospective case-control | AIS | 314 | 251 | 88 | N | 3months | >2 |
| 2 | Yuan Xie  2019 | Single center | retrospective case-control | AIS in the anterior circulation | 512 | Cross-Validation | | N | 3months | >2 |
| 3 | Hsueh-Lin Wang  2019 | Single center | retrospective case-control | ICH | 243 | Cross-Validation | | N | 6months | >2 |
| 4 | Hendrikus J. A. van Os 2018 | MR CLEAN study | retrospective case-control | AIS with EVT treatment | 1383 | Cross-validation | | N | 3months | >2 |
| 5 | Lucas A. Ramos  2020 | MR CLEAN study | retrospective case-control | AIS in the anterior circulation EVT | 1526 | Cross-validation | | N | 3months | ＞4 |
| 6 | Dougho Park  2021 | Single center | retrospective case-control | AIS | 1066 | 769 | 297 | N | 3months | >1 |
| 7 | Hidehisa Nishi  2020 | Multicenters | retrospective case-control | Large vessel AIS received MT | 324 | 250 | 74 | Y | 3months | >2 |
| 8 | Hidehisa Nishi  2019 | Multicenters | retrospective case-control | Large vessel AIS received MT | 502 | 387 | 115 | Y | 3months | >2 |
| 9 | Tomohisa Nezu  2022 | Multicenters | retrospective case-control | AIS | 1219 | Cross-Validation | | N | 3months | >2 |
| 10 | Eric Moulton  2019 | Single center | retrospective case-control | AIS with 4.5h thrombolysis | 87 | Repeated stratified nested cross-validation | | N | 3months | >2 |
| 11 | Xinping Lin  2021 | Single center | retrospective case-control | AIS | 1905 | 1524 | 381 | N | 3months | >1 |
| 12 | Ching-Heng Lin  2020 | TSR database | retrospective case-control | Ischemic stroke | 35798 | Cross-Validation | | N | 3months | >2 |
|  |  |  |  | Primary ICH | 4495 | Cross-Validation | | N | 3months | >2 |
| 13 | Yaru Liang  2019 | Single center | retrospective case-control | Ischemic stroke | 435 | 262 | 173 | N | 3months | >2 |
| 14 | Xiang Li  2020 | Single center | retrospective cohort study | AIS | 1735 | 1388 | 347 | N | 6months | >2 |
| 15 | Chulho Kim  2020 | Single center | retrospective case-control | AIS within 7days | 328 | 197 | 131 | N | 3months | >2 |
| 16 | B. Jiang  2021 | Single center | retrospective case-control | AIS | 1431 | Cross-Validation | | N | 3months | >2 |
| 17 | Hilbert A  2019 | MR CLEAN study | retrospective case-control | AIS with EVT | 1301 | Cross-Validation | | N | 3months | >2 |
| 18 | JoonNyung  2019 | Single center | retrospective case-control | AIS | 2604 | 1744 | 561 | N | 3months | >2 |
| 19 | Andrew N. Hall  2021 | Multicenters | retrospective cohort study | Acute ICH | 484 | Cross-Validation | | Y | 3months | ＞3 |
| 20 | Rui Guo  2022 | Single center | retrospective case-control | ICH | 751 | Cross-Validation | | N | 3months | >2 |
| 21 | Xiaobing Feng  2021 | Single center | retrospective case-control | AIS within 12 h | 499 | Repeated grouping was performed 3 times | | N | 6months | >2 |
| 22 | 1. Min Chiu   2021 | Single center | retrospective case-control | AIS with reperfusion ,EVT,  IVT | 590 | Cross-Validation | | N | 3months | ＞4 |
| 23 | Hung-Wen Chiu  2018 | Single center | retrospective case-control | AIS with IVT | 157 | 125 | 75 | N | 3months | >2 |
| 24 | Nai-Fang Chi  2021 | Single center | retrospective case-control | AIS | 150 | Cross-Validation | | N | 3months | >2 |
| 25 | Stephen Bacchi  2020 | Multicenters | retrospective case-control | AIS IVT | 204 | 173 | 91 | N | 3months | >1 |
| 26 | Ali alawieh  2019 | Single center | retrospective case-control | AIS patients with ADAPT  thrombectomy | 146 | Cross-Validation | | N | 3months | >2 |
| 27 | Shakiru A. Alaka  2020 | Multicenters | prospective cohort study | AIS with intravenous and/or intra arterial therapy | 1121 | 614 | 507 | Y | 3months | >2 |
| 28 | Thanh G. Phan  2017 | Single center | retrospective case-control | ICH | 957 | 638 | 319 | N | 3months | >2 |
| 29 | Zhang, X. G.  2022 | Single center | retrospective case-control | AIS with MT | 258 | NR | NR | N | 3months | >2 |
| 30 | Cheng Zhang  2022 | Single center | prospective cohort study | AIS | 93 | NR | NR | N | 3months | >2 |
| 31 | Zhelv Yao  2022 | Single center | retrospective case-control | AIS with MT | 217 | 163 | 52 | Y | 3months | >2 |
| 32 | Moulton, E.  2023 | Multicenters | retrospective case-control | AIS | 322 | Cross-Validation | | N | 3months | >2 |
| 33 | Ding, G. Y.  2022 | Single center | prospective cohort study | AIS | 132 | Bootstrapping | | N | 3months | >3 |
| 34 | Zhou, Y.  2022 | Single center | retrospective case-control | AIS | 522 | Cross-Validation | | Y | 6months | >2 |
| 35 | Qingqing Xu  2023 | Multicenters | retrospective case-control | AIS | 257 | 118 | 48 | Y | 3months | >2 |
| 36 | Tao, Z.  2023 | Single center | retrospective case-control | AIS | 65 | NR | NR | N | 3months | >2 |
| 37 | Ramos, L. A.  2022 | MR CLEAN study | prospective  cohort study | AIS with EVT | 3001 | Cross-Validation | | N | 3months | >2 |
| 38 | zhengping  2022 | Multicenters | retrospective case-control | AIS with IVT | 918 | NR | NR | Y | 3months | >1 |
| 39 | Jingwei Li  2022 | Multicenters | retrospective case-control | AIS | 1142 | 778 | 194 | Y | 3months | >2 |
| 40 | Jiawen Li  2022 | Multicenters | prospective  cohort study | ICH | 738 | 585 | NR | Y | 3months | >3 |
| 41 | Helge C. Kniep  2022 | Multicenters | retrospective case-control | Posterior circulation stroke | 172 | NR | NR | N | 3months | >2 |
| 42 | Mohamed Sobhi Jabal  2022 | Multicenters | retrospective case-control | AIS with thrombectomy | 293 | Cross-Validation | | N | 3months | >2 |
| 43 | Xiaoyu Huang  2022 | Multicenters | retrospective case-control | ICH | 1122 | 835 | 287 | N | 3months | >3 |
| 44 | Jin Hu  2022 | Multicenters | retrospective case-control | AIS with IVT | 849 | 594 | 255 | N | 3months | >2 |

EVT Endovascular thrombectomy

ADAPT A direct aspiration first pass technique

MT Mechanical thrombectomy

IVT Intravenous thrombolysis
