# Supplementary material for: Machine learning prediction of motor function in chronic stroke patients: a systematic review and meta-analysis
Source: Front Neurol. 2023 Jun 13;14:1039794. doi: 10.3389/fneur.2023.1039794 (PMC10299899; doi:10.3389/fneur.2023.1039794)
Supplement: Supplementary file 3 [file Table_3.DOCX]

Table S3 Models basic information

| NO. | First author | Year | Missing data processing method | Total patient number (n) | Predictors selection method | Model type |
| --- | --- | --- | --- | --- | --- | --- |
| 1 | Esra Zihni | 2020 | Mean+mode imputation | 314 | Zero-mean unit variance normalization | GLM\LASSO\ANN\Tree boosting \MLPs |
| 2 | Yuan Xie | 2019 | NA | 512 | Minimizing the mean squared error | XGB\GBM |
| 3 | Hsueh-Lin Wang | 2019 | SMOTE | 243 | Attribute Selection | RF |
| 4 | Hendrikus J. A. van Os | 2018 | Multiple imputations+Variables more than 25% missing values discarded | 1383 | Backward elimination  +Random Forests | RF\SVM\Neural Network\Super Learner |
| 5 | LucasA. Ramos | 2020 | Multiple imputations+single imputation approach using Random-Forest Imputation | 1526 | LASSO + Balanced class weights | RF\SVM\ANN\XGB\LR |
| 6 | Dougho Park | 2021 | Exclude | 1066 | Linear model+ Algorithms | RLR\SVM\RF\KNN\XGB |
| 7 | Hidehisa Nishi | 2020 | NA | 324 | NA | CNN |
| 8 | Hidehisa Nishi | 2019 | Data imputation of missing values was not performed | 502 | Univariate logistic regression  +Multiple logistic regression | RLR\SVM\RF\LR |
| 9 | Tomohisa Nezu | 2022 | NA | 1219 | Multivariable logistic analysis+  Backward selection procedure | LLGMN |
| 10 | Eric Moulton | 2019 | NA | 87 | NA | SVM |
| 11 | Xinping Lin | 2021 | K-nearest neighbor algorithm | 1905 | LASSO + SHAP | LR\SVM\RFC\XGB\DNN |
| 12 | Ching-Heng Lin | 2020 | NA | 40293 | Extremely randomized trees algorithm | SVM\RF\ANN\HANN |
| 13 | Yaru Liang | 2019 | NA | 435 | Multivariate logistic regression | ANN\MLR |
| 14 | Xiang Li | 2020 | Discard | 1735 | Univariable analysis+  Multivariable analysis +  Backward stepwise | LR\SVM\RFC/XGB/DNN |
| 15 | Chulho Kim | 2022 | NA | 328 | Extreme gradient boosting algorithm | XGB\SVM |
| 16 | B. Jiang, | 2021 | NA | 1431 | Relative weight | XGB |
| 17 | Hilbert A | 2019 | NA | 1301 | NA | RFNNs |
| 18 | JoonNyung | 2019 | NA | 2604 | NA | DNN\RF\LR |
| 19 | AndrewN. Hall | 2021 | Exclude | 484 | Decision tree-based algorithm | DT\RF |
| 20 | Rui Guo | 2022 | NA | 751 | Univariate analysis +  RFECV | LR\LRCV\SVM\RF\XGB\CatB |
| 21 | Xiaobing Feng | 2021 | Discard | 499 | Multivariate analysis+  Random forest | RF\XGB\ADB\SVM |
| 22 | 1. Min Chiu | 2021 | NA | 590 | Forward stepwise method | LR\SVM\RF\XGB |
| 23 | Hung-Wen Chiu | 2018 | NA | 157 | Logistic regression method | ANN |
| 24 | Nai-Fang Chi | 2021 | NA | 150 | Univariate analysis+  Wrapper method | Naıve Bayes\J48 \SMO\RF |
| 25 | Stephen Bacchi | 2020 | Median imputation | 204 | NA | ANN\CNN |
| 26 | ali alawieh | 2019 | NA | 146 | NA | Regression tree\DT |
| 27 | Shakiru A. Alaka | 2020 | Median imputation+  missing data more than 50% excluded | 1121 | Backward elimination+  Clinical expert knowledge+  Automated variable selection | RF/CRT/DT/SVM/ABM/LASSO/LR |
| 28 | Thanh G. Phan | 2017 | Missing data more than 10% excluded | 957 | Decision tree-based algorithms | Binary tree/Trichotomy tree |
| 29 | Zhang, X. G. | 2022 | NA | 258 | Lasso forward stepwise selection +  Multivariable logistic | LR |
| 30 | Cheng Zhang | 2022 | Multiple imputations | 93 | Least absolute shrinkage +  Selection operator regression | LR |
| 31 | Zhelv Yao | 2022 | NA | 217 | SHAP values | RF\ GB\ XGB\ CatBoost\ AdaBoost\ LightGBM\ extra trees |
| 32 | Moulton, E. | 2023 | NA | 322 | Attention mechanism | LR\CNN |
| 33 | Ding, G. Y. | 2022 | NA | 132 | Lasso+ Multivariable logistic | LR |
| 34 | Zhou, Y. | 2022 | Exclude | 522 | mRMR +  LASSO+  Backward stepdown selection procedure | LR |
| 35 | Qingqing Xu | 2023 | Exclude | 257 | mRMR +  LASSO | LR |
| 36 | Tao, Z. | 2023 | NA | 65 | Binomial Logistic regression +  SHAP | LR |
| 37 | Ramos, L. A. | 2022 | Multiple imputations | 3001 | SHAP | RF\ SVM\ NN\ XGB\LR |
| 38 | zhengping | 2022 | Exclude | 918 | Univariate logistic regression+  Backward stepdown selection | LR |
| 39 | Jingwei Li | 2022 | NA | 1142 | LightGBM algorithm assigned different weightings | LightGBM |
| 40 | Jiawen Li | 2022 | NA | 738 | Univariate+  Multivariable logistic  Backward stepwise elimination | LR |
| 41 | HelgeC. Kniep | 2022 | NA | 172 | Univariate+  Multivariable logistic | RF |
| 42 | Mohamed Sobhi Jabal | 2022 | NA | 293 | Univariate logistic | KNN\RF\GB\XGB |
| 43 | Xiaoyu Huang | 2022 | NA | 1122 | Univariate+  Multivariable logistic | LR |
| 44 | Jin Hu | 2022 | NA | 849 | Univariate+  Multivariable logistic | LR |

CNN Convolutional neural network

GLM Generalized Linear Model

HANN Hybrid artificial neural network

LASSO Least absolute selection and shrinkage operator

LLGMN Log-linearized Gaussian mixture network

LRCV Logistic regression cross-validation

mRMR Minimum redundancy and maximum

RFC Random forest classifier

RFECV Recursive feature elimination with cross-validation

RFNN Structured Receptive Fields

SHAP SHapley Additive exPlanations

SMO Sequential minimal optimization

SMOTE Synthetic minority oversampling technique
