# Supplementary material for: Machine learning prediction of motor function in chronic stroke patients: a systematic review and meta-analysis
Source: Front Neurol. 2023 Jun 13;14:1039794. doi: 10.3389/fneur.2023.1039794 (PMC10299899; doi:10.3389/fneur.2023.1039794)
Supplement: Supplementary file 4 [file Table_4.DOCX]

Table 4 Radiomics-based predictors machine learning models information

| First author and year | Model | Study characteristics | | | Predictor |
| --- | --- | --- | --- | --- | --- |
|  |  | Imaging method | Input parameters | Ground Truth |  |
| Hidehisa Nishi  2020 | 2-output deep learning model basedon convolutional neural networks. | MRI-DWI | Field strength\slice thickness\voxel size field of view\echo time\repetition time\bvalue\ADC maps | Labeling and Estimation of the Ischemic Core Lesion Each ischemic core lesion manually segmented on the native DWI performed by a single neurointerventionalist. | Imaging features |
| Eric Moulton  2019 | SVM classifiers built with axial diffusivity maps | MRI- DTI | Fractional Anisotropy  \MeanDiffusivity  \Axial Diffusivity  \Radial Diffusivity. | Lesion segmentation performed by identifying hypersignal regions on the 3-direction DWI sequence image and co-registered to the DTI maps. | Imaging features  +Clinical data |
| B. Jiang  2021 | XGB | CT | NECT(ASPECTS/HMCAS)CTA(Site  of occlusion\Collaterals\CTA-CBS)  NASCET (infarct side\collateral side)  CTP(Ischemic side\  Infarct core volume\Penumbra volume) | CTP datasets were processed on Brain Perfusion, Version 6.0.0; Philips Healthcare.Automatic segmentation of ischemic core and penumbra volumes was performed by blinded neuroradiologist. The site of occlusion、TIMI score, collateral status were interpreted on the MIP CTA images. | Radiological data  +Clinical data |
| Hilbert A  2019 | RFNNs | CT angiography | MIPs | Imaging data (CTA scans) available before EVT were used to develop the DL models and to determine radiological image biomarkers by expert radiologists. | Radiological data |
| Zhang, X. G.  2022 | LR | CT scan perfusion | TOAST\collateral circulation\vascular occlusion site\ mTICI score \ sICH | Occlusion of the site confrmed by digital subtraction angiography. | Radiological data  +Clinical data |
| Zhelv Yao  2022 | RF\GB\XGB\CatBoost\ADB\ LightGBM\ET | CT scan perfusion | ASPECTS\Collateralstatus\CBF\Tmax\mismatchvolume\mismat-ch ratio | The variables of PFCML-MT score comprised a range of domains, including demographic characteristics, clinical factors, laboratory indices, and radiological data. | Radiological data  +Clinical data |
| Moulton, E.  2023 | LR\  3D CNN classifiers with a trainable attention module | MRI-DWI | Pixel spacing\ slice thickness\slice gap\ number of slices \b-value | Small manual corrections were made to remove uninformative slices containing extra-cerebral tissue by a reader blinded to clinical data | Imaging features  +Clinical data |
| Zhou, Y.  2022 | LR | MRI-DWI | 3T MRI scanner repetition time\ echo time \ slice thickness\b value. For the 1.5T MRI scanner TR\ TE \slice thickness \ b value. | All DWI data were imported into the GE ADW 4.6 workstation.The AIS locations were classified by a neuroradiologist who was blinded to the patients’ clinical information according to the feeding artery of the infarction on DWI. | Imaging features  +Clinical data |
| Qingqing Xu  2023 | LR | DWI, FLAIR, and DWI-FLAIR | FLAIR [TR\TE\TI)], visual field (FOV), matrix, DWI (TR \TE)\FOV\ matrix\B value | All the scores were assessed by 2 experienced neuroradio logists. | Imaging features  +Clinical data |
| Tao, Z.  2023 | LR | CT, MRI-DWI | T2-FLAIR sequence: TR \TE \ FOV \ matrix \NAX \ layer thickness \spacing | Baseline CT image or DWI was used to grade the ASPECTS standardized 10-point scale by two-stroke neurologists.  Olindo scoring method was used to evaluate the FVH. | Imaging features+  +Clinical data |
| Ramos, L. A.  2022 | RF\SVM\LR\XGB\  ANN | CT angiography | Floating thrombus \ Pseudo-occlusion \ Carotid dissection \ Collateral score \Atherosclerotic occlusion on CTA baseline by core lab | Atlas as a reference scan, with a size of 256 × 256 × 90 voxels | Radiological data  +Clinical data |
| Helge C. Kniep  2022 | LR | NCCT images | 128-slice dual-source CT scanner with tube voltage\ tube current \ slice reconstruction\plane resolution iCT 256™ scanner tube voltage \ tube current \slice reconstruction \in plane resolution | Manual segmentations of the respective regions by experienced neuroradiologis on the original NCCT images using Analyze 11.0 Software. And transformed into standard space from image registration to the custom MNI-152 CT reference atlas. | Radiological data  +Clinical data |
| Mohamed Sobhi Jabal  2022 | KNN\RF\GBM\XGB | CT angiography | Non-acute infarct volume\total brain volume\ atrophy\ segmentation volumes e-CTA identifies large vessel occlusion site\ collateral circulation volume from e-CTA | Quantitative imaging feature extraction was performed using e-Stroke software for automated calculation of ASPE CT Score and estimated acute infarct volumes on NCCT. | Imaging features+  Clinical data |

ADC Apparent diffusion coefficient

DTI MRI-Diffusion Tensor Imaging

NECT Non-contrast-enhanced CT

mTICI Modifed Trombolysis in Cerebral Infraction

sICH Symptomatic intracranial hemorrhage

CBF Cerebral blood flow

FVH Vascular hyperintensities

TI Inversion time

TIMI Thrombolysis in Myocardial Infarction score

MIPs Maximum Intensity Projections

NCCT Non-contrast CT
