# Supplementary material for: Machine learning prediction of motor function in chronic stroke patients: a systematic review and meta-analysis
Source: Front Neurol. 2023 Jun 13;14:1039794. doi: 10.3389/fneur.2023.1039794 (PMC10299899; doi:10.3389/fneur.2023.1039794)
Supplement: Supplementary file 5 [file Table_5.DOCX]

##### Checklists from Prediction model Risk of Bias Assessment Tool (PROBAST)^[1]^.

| **DOMAIN 1: Participants** | |
| --- | --- |
| **A. Risk of Bias** | |
| - 1. Were appropriate data sources used, e.g. cohort, RCT or nested case-control study data? |  |
| - 1. Were all inclusions and exclusions of participants appropriate? |  |
| **Risk of bias introduced by selection of participants** | **RISK:**  *(low/ high/ unclear)* |

| **DOMAIN 2: Predictors** | |
| --- | --- |
| **A. Risk of Bias** | |
| - 1. Were predictors defined and assessed in a similar way for all participants? | |
| - 1. Were predictor assessments made without knowledge of outcome data? | |
| - 1. Are all predictors available at the time the model is intended to be used? | |
| **Risk of bias introduced by predictors or their assessment** | **RISK:**  *(low/ high/ unclear)* |

| **DOMAIN 3: Outcome** | | | |
| --- | --- | --- | --- |
| **Risk of Bias** | | | |
| - 1. Was the outcome determined appropriately? | | | |
| - 1. Was a pre-specified or standard outcome definition used? | | | |
| - 1. Were predictors excluded from the outcome definition? | | | |
| - 1. Was the outcome defined and determined in a similar way for all participants? | | | |
| - 1. Was the outcome determined without knowledge of predictor information? | | | |
| - 1. Was the time interval between predictor assessment and outcome determination appropriate? | | | |
| **Risk of bias introduced by outcome** | | **RISK:**  *(low/ high/ unclear)* | |
| **DOMAIN 4: Analysis** | | |  |
| **Risk of Bias** | | |  |
| - 1. Were there a reasonable number of participants with the outcome? i.e. Was the sample size adequately powered? | | |  |
| - 1. Were continuous and categorical predictors handled appropriately? | | |  |
| - 1. Were all enrolled participants included in the analysis? | | |  |
| - 1. Were participants with missing data handled appropriately? | | |  |
| - 1. Was selection of predictors based on univariable analysis avoided? | | |  |
| - 1. Were complexities in the data accounted for appropriately? | | |  |
| - 1. Were relevant model performance measures evaluated appropriately? | | |  |
| - 1. Were model overfitting and optimism in model performance accounted for? | | |  |
| - 1. Do predictors and their assigned weights in the final model correspond to the results from the reported multivariable analysis? | | |  |
| **Risk of bias introduced by outcome** | **RISK:**  *(low/ high/ unclear)* | |  |

[1]Moons KGM, Wolff RF, Riley RD, Whiting PF, Westwood M, Collins GS, Reitsma JB, Kleijnen J, Mallett S. PROBAST: A Tool to Assess Risk of Bias and Applicability

of Prediction Model Studies: Explanation and Elaboration. Ann Intern Med. 2019 Jan 1;170(1):W1-W33.

| No | Author | Year | Participants | | Predictors | | | Outcome | | | | | | Analysis | | | | | | | | |
| --- | --- | --- | --- | --- | --- | --- | --- | --- | --- | --- | --- | --- | --- | --- | --- | --- | --- | --- | --- | --- | --- | --- |
|  |  |  | question  1 | question  2 | question1 | question  2 | question3 | question1 | question2 | question  3 | question4 | question5 | question  6 | question  1 | question2 | question3 | question4 | question5 | question6 | question7 | question8 | question9 |
| 1 | Esra Zihni | 2020 | High | Low | Low | Unclear | Low | Low | Low | Low | Low | Unclear | Low | High | Low | Low | High | Unclear | Unclear | Low | Low | Low |
| 1 | Esra Zihni | 2020 | High | Low | Low | Unclear | Low | Low | Low | Low | Low | Unclear | Low | High | Low | Low | High | Unclear | Unclear | Low | Low | Low |
| 1 | Esra Zihni | 2020 | High | Low | Low | Unclear | Low | Low | Low | Low | Low | Unclear | Low | High | Low | Low | High | Unclear | Unclear | Low | Low | Low |
| 1 | Esra Zihni | 2020 | High | Low | Low | Unclear | Low | Low | Low | Low | Low | Unclear | Low | High | Low | Low | High | Unclear | Unclear | Low | Low | Low |
| 1 | Esra Zihni | 2020 | High | Low | Low | Unclear | Low | Low | Low | Low | Low | Unclear | Low | High | Low | Low | High | Unclear | Unclear | Low | Low | Low |
| 2 | Yuan Xie | 2019 | High | Low | Low | Unclear | Unclear | Low | Low | Low | Low | Unclear | Low | Low | Low | Low | Unclear | Unclear | Unclear | Low | Low | Low |
| 2 | Yuan Xie | 2019 | High | Low | Low | Unclear | Unclear | Low | Low | Low | Low | Unclear | Low | Low | Low | Low | Unclear | Unclear | Unclear | Low | Low | Low |
| 3 | Hsueh-Lin Wang | 2019 | High | Low | Low | Unclear | Low | Low | Low | Low | Low | Unclear | Low | High | Low | Low | Low | Low | Unclear | Low | Low | Low |
| 4 | Hendrikus J. A. van Os | 2018 | Low | Low | Low | Low | High | Low | Low | Low | Low | Unclear | Low | High | Low | Low | Low | Low | Unclear | Low | Low | Low |
| 4 | Hendrikus J. A. van Os | 2018 | Low | Low | Low | Low | High | Low | Low | Low | Low | Unclear | Low | High | Low | Low | Low | Low | Unclear | Low | Low | Low |
| 4 | Hendrikus J. A. van Os | 2018 | Low | Low | Low | Low | High | Low | Low | Low | Low | Unclear | Low | High | Low | Low | Low | Low | Unclear | Low | Low | Low |
| 4 | Hendrikus J. A. van Os | 2018 | Low | Low | Low | Low | High | Low | Low | Low | Low | Unclear | Low | High | Low | Low | Low | Low | Unclear | Low | Low | Low |
| 5 | Lucas A. Ramos | 2020 | Low | Low | Low | Low | Low | High | Low | Low | Low | Unclear | Low | Low | Low | Low | Low | Low | Unclear | Low | Low | Low |
| 5 | Lucas A. Ramos | 2020 | Low | Low | Low | Low | Low | High | Low | Low | Low | Unclear | Low | Low | Low | Low | Low | Low | Unclear | Low | Low | Low |
| 5 | Lucas A. Ramos | 2020 | Low | Low | Low | Low | Low | High | Low | Low | Low | Unclear | Low | Low | Low | Low | Low | Low | Unclear | Low | Low | Low |
| 5 | Lucas A. Ramos | 2020 | Low | Low | Low | Low | Low | High | Low | Low | Low | Unclear | Low | Low | Low | Low | Low | Low | Unclear | Low | Low | Low |
| 5 | Lucas A. Ramos | 2020 | Low | Low | Low | Low | Low | High | Low | Low | Low | Unclear | Low | Low | Low | Low | Low | Low | Unclear | Low | Low | Low |
| 6 | Dougho Park | 2021 | High | Low | Low | High | Low | Low | Low | Low | Low | Low | Low | Low | Low | Low | High | Unclear | Unclear | Low | Low | Low |
| 6 | Dougho Park | 2021 | High | Low | Low | High | Low | Low | Low | Low | Low | Low | Low | Low | Low | Low | High | Unclear | Unclear | Low | Low | Low |
| 6 | Dougho Park | 2021 | High | Low | Low | High | Low | Low | Low | Low | Low | Low | Low | Low | Low | Low | High | Unclear | Unclear | Low | Low | Low |
| 6 | Dougho Park | 2021 | High | Low | Low | High | Low | Low | Low | Low | Low | Low | Low | Low | Low | Low | High | Unclear | Unclear | Low | Low | Low |
| 6 | Dougho Park | 2021 | High | Low | Low | High | Low | Low | Low | Low | Low | Low | Low | Low | Low | Low | High | Unclear | Unclear | Low | Low | Low |
| 7 | Hidehisa Nishi | 2020 | High | Low | Low | High | Unclear | Low | Low | Low | Low | Low | Low | Low | Low | Low | High | Unclear | Unclear | Low | Low | Low |
| 8 | Hidehisa Nishi | 2019 | High | Low | Low | High | Unclear | Low | Low | Low | Low | Low | Low | Low | Low | Low | High | Low | Unclear | Low | Low | Low |
| 8 | Hidehisa Nishi | 2019 | High | Low | Low | High | Unclear | Low | Low | Low | Low | Low | Low | Low | Low | Low | High | Low | Unclear | Low | Low | Low |
| 8 | Hidehisa Nishi | 2019 | High | Low | Low | High | Unclear | Low | Low | Low | Low | Low | Low | Low | Low | Low | High | Low | Unclear | Low | Low | Low |
| 8 | Hidehisa Nishi | 2019 | High | Low | Low | High | Unclear | Low | Low | Low | Low | Low | Low | Low | Low | Low | High | Low | Unclear | Low | Low | Low |
| 9 | Tomohisa Nezu | 2022 | High | Low | Low | High | Unclear | Low | Low | Low | Low | Low | Low | Low | Low | Low | Unclear | Low | Unclear | Low | Low | Low |
| 9 | Tomohisa Nezu | 2022 | High | Low | Low | High | Unclear | Low | Low | Low | Low | Low | Low | Low | Low | Low | Unclear | Low | Unclear | Low | Low | Low |
| 10 | Eric Moulton | 2019 | High | Low | Low | High | Unclear | Low | Low | Low | Low | Unclear | Low | High | Low | Low | Unclear | Low | Unclear | Low | Low | Low |
| 10 | Eric Moulton | 2019 | High | Low | Low | High | Unclear | Low | Low | Low | Low | Unclear | Low | High | Low | Low | Unclear | Low | Unclear | Low | Low | Low |
| 10 | Eric Moulton | 2019 | High | Low | Low | High | Unclear | Low | Low | Low | Low | Unclear | Low | High | Low | Low | Unclear | Low | Unclear | Low | Low | Low |
| 10 | Eric Moulton | 2019 | High | Low | Low | High | Unclear | Low | Low | Low | Low | Unclear | Low | High | Low | Low | Unclear | Low | Unclear | Low | Low | Low |
| 10 | Eric Moulton | 2019 | High | Low | Low | High | Unclear | Low | Low | Low | Low | Unclear | Low | High | Low | Low | Unclear | Low | Unclear | Low | Low | Low |
| 11 | Xinping Lin | 2021 | High | Low | Low | High | High | Low | Low | Low | Low | Unclear | Low | Low | Low | Low | Low | Low | Unclear | Low | Low | Low |
| 11 | Xinping Lin | 2021 | High | Low | Low | High | High | Low | Low | Low | Low | Unclear | Low | Low | Low | Low | Low | Low | Unclear | Low | Low | Low |
| 11 | Xinping Lin | 2021 | High | Low | Low | High | High | Low | Low | Low | Low | Unclear | Low | Low | Low | Low | Low | Low | Unclear | Low | Low | Low |
| 11 | Xinping Lin | 2021 | High | Low | Low | High | High | Low | Low | Low | Low | Unclear | Low | Low | Low | Low | Low | Low | Unclear | Low | Low | Low |
| 11 | Xinping Lin | 2021 | High | Low | Low | High | High | Low | Low | Low | Low | Unclear | Low | Low | Low | Low | Low | Low | Unclear | Low | Low | Low |
| 12 | Ching-Heng Lin | 2020 | Low | Low | Low | Low | Unclear | Low | Low | Low | Low | Low | Low | Low | Low | Low | Low | Low | Unclear | Low | Low | Low |
| 12 | Ching-Heng Lin | 2020 | Low | Low | Low | Low | Unclear | Low | Low | Low | Low | Low | Low | Low | Low | Low | Low | Low | Unclear | Low | Low | Low |
| 12 | Ching-Heng Lin | 2020 | Low | Low | Low | Low | Unclear | Low | Low | Low | Low | Low | Low | Low | Low | Low | Low | Low | Unclear | Low | Low | Low |
| 12 | Ching-Heng Lin | 2020 | Low | Low | Low | Low | Unclear | Low | Low | Low | Low | Low | Low | Low | Low | Low | Low | Low | Unclear | Low | Low | Low |
| 12 | Ching-Heng Lin | 2020 | Low | Low | Low | Low | Unclear | Low | Low | Low | Low | Low | Low | Low | Low | Low | Low | Low | Unclear | Low | Low | Low |
| 12 | Ching-Heng Lin | 2020 | Low | Low | Low | Low | Unclear | Low | Low | Low | Low | Low | Low | Low | Low | Low | Low | Low | Unclear | Low | Low | Low |
| 12 | Ching-Heng Lin | 2020 | Low | Low | Low | Low | Unclear | Low | Low | Low | Low | Low | Low | Low | Low | Low | Low | Low | Unclear | Low | Low | Low |
| 12 | Ching-Heng Lin | 2020 | Low | Low | Low | Low | Unclear | Low | Low | Low | Low | Low | Low | Low | Low | Low | Low | Low | Unclear | Low | Low | Low |
| 13 | Yaru Liang | 2019 | Low | Low | Low | Low | Unclear | Low | Low | Low | Low | Low | Low | Low | Low | Low | Unclear | Low | Unclear | Low | Unclear | Low |
| 13 | Yaru Liang | 2019 | Low | Low | Low | Low | Unclear | Low | Low | Low | Low | Low | Low | Low | Low | Low | Unclear | Low | Unclear | Low | Unclear | Low |
| 14 | Xiang Li | 2020 | High | Low | Low | Low | Low | Low | Low | Low | Low | Low | Low | Low | Low | Low | High | Low | Unclear | Low | Low | Low |
| 14 | Xiang Li | 2020 | High | Low | Low | Low | Low | Low | Low | Low | Low | Low | Low | Low | Low | Low | High | Low | Unclear | Low | Low | Low |
| 14 | Xiang Li | 2020 | High | Low | Low | Low | Low | Low | Low | Low | Low | Low | Low | Low | Low | Low | High | Low | Unclear | Low | Low | Low |
| 14 | Xiang Li | 2020 | High | Low | Low | Low | Low | Low | Low | Low | Low | Low | Low | Low | Low | Low | High | Low | Unclear | Low | Low | Low |
| 14 | Xiang Li | 2020 | High | Low | Low | Low | Low | Low | Low | Low | Low | Low | Low | Low | Low | Low | High | Low | Unclear | Low | Low | Low |
| 15 | Chulho Kim | 2022 | High | Low | Low | High | Low | Low | Low | Low | Low | Low | Low | High | Low | Low | Unclear | Low | Unclear | Low | Low | Low |
| 15 | Chulho Kim | 2022 | High | Low | Low | High | Low | Low | Low | Low | Low | Low | Low | High | Low | Low | Unclear | Low | Unclear | Low | Low | Low |
| 16 | B. Jiang | 2021 | High | Low | Low | High | Unclear | Low | Low | Low | Low | Low | Low | Low | Low | Low | Unclear | Low | Unclear | Low | Unclear | Low |
| 17 | Hilbert A | 2019 | Low | Low | Low | Low | Unclear | Low | Low | Low | Low | Low | Low | Low | Low | Low | Unclear | Unclear | Unclear | Low | Low | Low |
| 18 | JoonNyung | 2019 | High | Low | Low | High | Unclear | Low | Low | Low | Low | Low | Low | Low | Low | Low | Unclear | Low | Unclear | Low | Low | Low |
| 18 | JoonNyung | 2019 | High | Low | Low | High | Unclear | Low | Low | Low | Low | Low | Low | Low | Low | Low | Unclear | Low | Unclear | Low | Low | Low |
| 18 | JoonNyung | 2019 | High | Low | Low | High | Unclear | Low | Low | Low | Low | Low | Low | Low | Low | Low | Unclear | Low | Unclear | Low | Low | Low |
| 19 | Andrew N. Hall | 2021 | Low | Low | Low | Low | Unclear | Low | Low | Low | Low | Low | Low | Low | Low | Low | High | Low | Unclear | Low | Low | Low |
| 19 | Andrew N. Hall | 2021 | Low | Low | Low | Low | Unclear | Low | Low | Low | Low | Low | Low | Low | Low | Low | High | Low | Unclear | Low | Low | Low |
| 20 | Rui Guo | 2022 | High | Low | Low | High | Unclear | Low | Low | Low | Low | Low | Low | Low | Low | Low | Unclear | Low | Unclear | Low | Unclear | Low |
| 20 | Rui Guo | 2022 | High | Low | Low | High | Unclear | Low | Low | Low | Low | Low | Low | Low | Low | Low | Unclear | Low | Unclear | Low | Unclear | Low |
| 20 | Rui Guo | 2022 | High | Low | Low | High | Unclear | Low | Low | Low | Low | Low | Low | Low | Low | Low | Unclear | Low | Unclear | Low | Unclear | Low |
| 20 | Rui Guo | 2022 | High | Low | Low | High | Unclear | Low | Low | Low | Low | Low | Low | Low | Low | Low | Unclear | Low | Unclear | Low | Unclear | Low |
| 20 | Rui Guo | 2022 | High | Low | Low | High | Unclear | Low | Low | Low | Low | Low | Low | Low | Low | Low | Unclear | Low | Unclear | Low | Unclear | Low |
| 20 | Rui Guo | 2022 | High | Low | Low | High | Unclear | Low | Low | Low | Low | Low | Low | Low | Low | Low | Unclear | Low | Unclear | Low | Unclear | Low |
| 21 | Xiaobing Feng | 2021 | High | Low | Low | High | Unclear | Low | Low | Low | Low | Low | Low | Low | Low | Low | High | Low | Unclear | Low | Unclear | Low |
| 21 | Xiaobing Feng | 2021 | High | Low | Low | High | Unclear | Low | Low | Low | Low | Low | Low | Low | Low | Low | High | Low | Unclear | Low | Unclear | Low |
| 21 | Xiaobing Feng | 2021 | High | Low | Low | High | Unclear | Low | Low | Low | Low | Low | Low | Low | Low | Low | High | Low | Unclear | Low | Unclear | Low |
| 21 | Xiaobing Feng | 2021 | High | Low | Low | High | Unclear | Low | Low | Low | Low | Low | Low | Low | Low | Low | High | Low | Unclear | Low | Unclear | Low |
| 22 | I-Min Chiu | 2021 | High | Low | Low | High | Unclear | Low | Low | Low | Low | Low | Low | High | Low | Low | Unclear | Low | Unclear | Low | Low | Low |
| 22 | I-Min Chiu | 2021 | High | Low | Low | High | Unclear | Low | Low | Low | Low | Low | Low | High | Low | Low | Unclear | Low | Unclear | Low | Low | Low |
| 22 | I-Min Chiu | 2021 | High | Low | Low | High | Unclear | Low | Low | Low | Low | Low | Low | High | Low | Low | Unclear | Low | Unclear | Low | Low | Low |
| 22 | I-Min Chiu | 2021 | High | Low | Low | High | Unclear | Low | Low | Low | Low | Low | Low | High | Low | Low | Unclear | Low | Unclear | Low | Low | Low |
| 23 | Hung-Wen Chiu | 2018 | High | Low | Low | High | Unclear | Low | Low | Low | Low | Low | Low | High | Low | Low | Unclear | Unclear | Unclear | Low | Unclear | Low |
| 24 | Nai-Fang Chi | 2021 | High | Low | Low | High | Unclear | Low | Low | Low | Low | Low | Low | High | Low | Low | Unclear | Low | Unclear | Low | Unclear | Low |
| 24 | Nai-Fang Chi | 2021 | High | Low | Low | High | Unclear | Low | Low | Low | Low | Low | Low | High | Low | Low | Unclear | Low | Unclear | Low | Unclear | Low |
| 24 | Nai-Fang Chi | 2021 | High | Low | Low | High | Unclear | Low | Low | Low | Low | Low | Low | High | Low | Low | Unclear | Low | Unclear | Low | Unclear | Low |
| 24 | Nai-Fang Chi | 2021 | High | Low | Low | High | Unclear | Low | Low | Low | Low | Low | Low | High | Low | Low | Unclear | Low | Unclear | Low | Unclear | Low |
| 25 | Stephen Bacchi | 2020 | High | Low | Low | High | Low | Low | Low | Low | Low | Low | Low | High | Low | Low | High | Unclear | Unclear | Low | Low | Low |
| 25 | Stephen Bacchi | 2020 | High | Low | Low | High | Low | Low | Low | Low | Low | Low | Low | High | Low | Low | High | Unclear | Unclear | Low | Low | Low |
| 25 | Stephen Bacchi | 2020 | High | Low | Low | High | Low | Low | Low | Low | Low | Low | Low | High | Low | Low | High | Unclear | Unclear | Low | Low | Low |
| 26 | ali alawieh | 2019 | High | Low | Low | High | Low | Low | Low | Low | Low | Low | Low | High | Low | Low | Unclear | Unclear | Unclear | Low | Low | Low |
| 26 | ali alawieh | 2019 | High | Low | Low | High | Low | Low | Low | Low | Low | Low | Low | High | Low | Low | Unclear | Unclear | Unclear | Low | Low | Low |
| 26 | ali alawieh | 2019 | High | Low | Low | High | Low | Low | Low | Low | Low | Low | Low | High | Low | Low | Unclear | Unclear | Unclear | Low | Low | Low |
| 26 | ali alawieh | 2019 | High | Low | Low | High | Low | Low | Low | Low | Low | Low | Low | High | Low | Low | Unclear | Unclear | Unclear | Low | Low | Low |
| 26 | ali alawieh | 2019 | High | Low | Low | High | Low | Low | Low | Low | Low | Low | Low | High | Low | Low | Unclear | Unclear | Unclear | Low | Low | Low |
| 26 | ali alawieh | 2019 | High | Low | Low | High | Low | Low | Low | Low | Low | Low | Low | High | Low | Low | Unclear | Unclear | Unclear | Low | Low | Low |
| 26 | ali alawieh | 2019 | High | Low | Low | High | Low | Low | Low | Low | Low | Low | Low | High | Low | Low | Unclear | Unclear | Unclear | Low | Low | Low |
| 26 | ali alawieh | 2019 | High | Low | Low | High | Low | Low | Low | Low | Low | Low | Low | High | Low | Low | Unclear | Unclear | Unclear | Low | Low | Low |
| 26 | ali alawieh | 2019 | High | Low | Low | High | Low | Low | Low | Low | Low | Low | Low | High | Low | Low | Unclear | Unclear | Unclear | Low | Low | Low |
| 26 | ali alawieh | 2019 | High | Low | Low | High | Low | Low | Low | Low | Low | Low | Low | High | Low | Low | Unclear | Unclear | Unclear | Low | Low | Low |
| 27 | Shakiru A. Alaka | 2020 | Low | Low | Low | Low | Low | Low | Low | Low | Low | Low | Low | Low | Low | Low | High | Low | Unclear | Low | Low | Low |
| 27 | Shakiru A. Alaka | 2020 | Low | Low | Low | Low | Low | Low | Low | Low | Low | Low | Low | Low | Low | Low | High | Low | Unclear | Low | Low | Low |
| 27 | Shakiru A. Alaka | 2020 | Low | Low | Low | Low | Low | Low | Low | Low | Low | Low | Low | Low | Low | Low | High | Low | Unclear | Low | Low | Low |
| 27 | Shakiru A. Alaka | 2020 | Low | Low | Low | Low | Low | Low | Low | Low | Low | Low | Low | Low | Low | Low | High | Low | Unclear | Low | Low | Low |
| 27 | Shakiru A. Alaka | 2020 | Low | Low | Low | Low | Low | Low | Low | Low | Low | Low | Low | Low | Low | Low | High | Low | Unclear | Low | Low | Low |
| 27 | Shakiru A. Alaka | 2020 | Low | Low | Low | Low | Low | Low | Low | Low | Low | Low | Low | Low | Low | Low | High | Low | Unclear | Low | Low | Low |
| 27 | Shakiru A. Alaka | 2020 | Low | Low | Low | Low | Low | Low | Low | Low | Low | Low | Low | Low | Low | Low | High | Low | Unclear | Low | Low | Low |
| 28 | Thanh G. Phan | 2017 | High | Low | Low | High | Low | Low | Low | Low | Low | Low | Low | Low | Low | Low | Unclear | Low | Unclear | Low | Unclear | Low |
| 28 | Thanh G. Phan | 2017 | High | Low | Low | High | Low | Low | Low | Low | Low | Low | Low | Low | Low | Low | Unclear | Low | Unclear | Low | Unclear | Low |
| 29 | Zhang, X. G. | 2022 | High | Low | Low | High | Unclear | Low | Low | Low | Low | Low | Low | High | Low | Low | Unclear | Low | Unclear | Low | Low | Low |
| 30 | Cheng Zhang | 2022 | Low | Low | Low | Low | Unclear | Low | Low | Low | Low | Low | Low | High | Low | Low | Low | Low | Unclear | Low | Low | Low |
| 31 | Zhelv Yao | 2022 | High | Low | Low | Low | Unclear | Low | Low | Low | Low | Low | Low | High | Low | Low | Unclear | Low | Unclear | Low | Low | Low |
| 31 | Zhelv Yao | 2022 | High | Low | Low | Low | Unclear | Low | Low | Low | Low | Low | Low | High | Low | Low | Unclear | Low | Unclear | Low | Low | Low |
| 31 | Zhelv Yao | 2022 | High | Low | Low | Low | Unclear | Low | Low | Low | Low | Low | Low | High | Low | Low | Unclear | Low | Unclear | Low | Low | Low |
| 31 | Zhelv Yao | 2022 | High | Low | Low | Low | Unclear | Low | Low | Low | Low | Low | Low | High | Low | Low | Unclear | Low | Unclear | Low | Low | Low |
| 31 | Zhelv Yao | 2022 | High | Low | Low | Low | Unclear | Low | Low | Low | Low | Low | Low | High | Low | Low | Unclear | Low | Unclear | Low | Low | Low |
| 31 | Zhelv Yao | 2022 | High | Low | Low | Low | Unclear | Low | Low | Low | Low | Low | Low | High | Low | Low | Unclear | Low | Unclear | Low | Low | Low |
| 31 | Zhelv Yao | 2022 | High | Low | Low | Low | Unclear | Low | Low | Low | Low | Low | Low | High | Low | Low | Unclear | Low | Unclear | Low | Low | Low |
| 32 | Moulton, E. | 2023 | High | Low | Low | Unclear | Unclear | Low | Low | Low | Low | Low | Low | High | Low | Low | Unclear | Unclear | Unclear | Low | Low | Low |
| 32 | Moulton, E. | 2023 | High | Low | Low | Unclear | Unclear | Low | Low | Low | Low | Low | Low | High | Low | Low | Unclear | Unclear | Unclear | Low | Low | Low |
| 33 | Ding, G. Y. | 2022 | Low | Low | Low | Low | Unclear | Low | Low | Low | Low | Low | Low | High | Low | Low | Unclear | Low | Unclear | Low | Low | Low |
| 34 | Zhou, Y. | 2022 | High | Low | Low | Unclear | Unclear | Low | Low | Low | Low | Low | Low | Low | Low | Low | Unclear | Low | Unclear | Low | Low | Low |
| 35 | Qingqing Xu | 2023 | High | Low | Low | Unclear | Unclear | Low | Low | Low | Low | Low | Low | High | Low | Low | Unclear | Low | Unclear | Low | Low | Low |
| 36 | Tao, Z. | 2023 | High | Low | Low | Unclear | Unclear | Low | Low | Low | Low | Low | Low | High | Low | Low | Unclear | Low | Unclear | Low | Low | Low |
| 36 | Tao, Z. | 2023 | High | Low | Low | Unclear | Unclear | Low | Low | Low | Low | Low | Low | High | Low | Low | Unclear | Low | Unclear | Low | Low | Low |
| 36 | Tao, Z. | 2023 | High | Low | Low | Unclear | Unclear | Low | Low | Low | Low | Low | Low | High | Low | Low | Unclear | Low | Unclear | Low | Low | Low |
| 37 | Ramos, L. A. | 2022 | Low | Low | Low | Low | Unclear | Low | Low | Low | Low | Low | Low | Low | Low | Low | Low | Low | Low | Low | Low | Low |
| 37 | Ramos, L. A. | 2022 | Low | Low | Low | Low | Unclear | Low | Low | Low | Low | Low | Low | Low | Low | Low | Low | Low | Low | Low | Low | Low |
| 37 | Ramos, L. A. | 2022 | Low | Low | Low | Low | Unclear | Low | Low | Low | Low | Low | Low | Low | Low | Low | Low | Low | Low | Low | Low | Low |
| 37 | Ramos, L. A. | 2022 | Low | Low | Low | Low | Unclear | Low | Low | Low | Low | Low | Low | Low | Low | Low | Low | Low | Low | Low | Low | Low |
| 37 | Ramos, L. A. | 2022 | Low | Low | Low | Low | Unclear | Low | Low | Low | Low | Low | Low | Low | Low | Low | Low | Low | Low | Low | Low | Low |
| 38 | zhengping | 2022 | High | Low | Low | Unclear | Unclear | Low | Low | Low | Low | Low | Low | Low | Low | Low | Unclear | Unclear | Unclear | Low | Low | Low |
| 39 | Jingwei Li | 2022 | High | Low | Low | Unclear | Unclear | Low | Low | Low | Low | Low | Low | Low | Low | Low | Unclear | Unclear | Unclear | Low | Low | Low |
| 40 | Jiawen Li | 2022 | Low | Low | Low | Low | Unclear | Low | Low | Low | Low | Low | Low | Low | Low | Low | Unclear | Low | Unclear | Low | Low | Low |
| 41 | Helge C. Kniep | 2022 | High | Low | Low | Unclear | Unclear | Low | Low | Low | Low | Low | Low | High | Low | Low | Unclear | Low | Unclear | Low | Low | Low |
| 42 | Mohamed Sobhi Jabal | 2022 | High | Low | Low | Unclear | Unclear | Low | Low | Low | Low | Low | Low | High | Low | Low | Unclear | Unclear | Unclear | Low | Low | Low |
| 42 | Mohamed Sobhi Jabal | 2022 | High | Low | Low | Unclear | Unclear | Low | Low | Low | Low | Low | Low | High | Low | Low | Unclear | Unclear | Unclear | Low | Low | Low |
| 42 | Mohamed Sobhi Jabal | 2022 | High | Low | Low | Unclear | Unclear | Low | Low | Low | Low | Low | Low | High | Low | Low | Unclear | Unclear | Unclear | Low | Low | Low |
| 42 | Mohamed Sobhi Jabal | 2022 | High | Low | Low | Unclear | Unclear | Low | Low | Low | Low | Low | Low | High | Low | Low | Unclear | Unclear | Unclear | Low | Low | Low |
| 43 | Xiaoyu Huang | 2022 | High | Low | Low | Unclear | Unclear | Low | Low | Low | Low | Low | Low | Low | Low | Low | Unclear | Low | Unclear | Low | Low | Low |
| 44 | Jin Hu | 2022 | High | Low | Low | Unclear | Unclear | Low | Low | Low | Low | Low | Low | Low | Low | Low | Unclear | Low | Unclear | Low | Low | Low |
